# Assemblies of amyloid-β_30–36_ hexamer and its G33V/L34T mutants by replica-exchange molecular dynamics simulation

**DOI:** 10.1371/journal.pone.0188794

**Published:** 2017-11-29

**Authors:** Zhenyu Qian, Qingwen Zhang, Yu Liu, Peijie Chen

**Affiliations:** 1 Key Laboratory of Exercise and Health Sciences (Ministry of Education) and School of Kinesiology, Shanghai University of Sport, Shanghai, China; 2 College of Physical Education and Training, Shanghai University of Sport, Shanghai, China; INRA Centre de Jouy-en-Josas, FRANCE

## Abstract

The aggregation of amyloid-β peptides is associated with the pathogenesis of Alzheimer’s disease, in which the 30–36 fragments play an important part as a fiber-forming hydrophobic region. The fibrillar structure of Aβ_30–36_ has been detected by means of X-ray diffraction, but its oligomeric structural determination, biophysical characterization, and pathological mechanism remain elusive. In this study, we have investigated the structures of Aβ_30–36_ hexamer as well as its G33V and L34T mutants in explicit water environment using replica-exchange molecular dynamics (REMD) simulations. Our results show that the wild-type (WT) Aβ_30–36_ hexamer has a preference to form β-barrel and bilayer β-sheet conformations, while the G33V or L34T mutation disrupts the β-barrel structures: the G33V mutant is homogenized to adopt β-sheet-rich bilayers, and the structures of L34T mutant on the contrary get more diverse. The hydrophobic interaction plays a critical role in the formation and stability of oligomeric assemblies among all the three systems. In addition, the substitution of G33 by V reduces the β-sheet content in the most populated conformations of Aβ_30–36_ oligomers through a steric effect. The L34T mutation disturbs the interpeptide hydrogen bonding network, and results in the increased coil content and morphological diversity. Our REMD runs provide structural details of WT and G33V/L34T mutant Aβ_30–36_ oligomers, and molecular insight into the aggregation mechanism, which will be helpful for designing novel inhibitors or amyloid-based materials.

## Introduction

Alzheimer’s disease (AD), characterized by cerebral extracellular amyloid plaques, is age-related and quite common among the senior population. Its pathogenesis is associated with the accumulation of amyloid β-peptide (Aβ) and τ-protein [1,2], and evidences from genetics and pathology support that the former trigger the pathogenesis process [3,4]. Soluble Aβmonomer is mainly disordered, while the major constituents of amyloid plaques display a cross-β structure [5,6]. The mature amyloid fibril is believed to associate with neurologic degeneration [2]. However, converging studies suggest that small intermediate oligomers are the major species responsible for neurotoxicity and dementia, which are formed in the early stage of aggregation [7,8].

The Aβ peptide (40–42−amino acid) is the most abundant forms of Aβ *in vivo*, derived from the amyloid precursor protein (APP) through proteolytic cleavage by β- and γ-secretase [9]. In 2005, a β1-strand–turn–β2-strand motif was proposed to describe the Aβ_1–42_ fibrillar structure using nuclear magnetic resonance (NMR), which contains two in-register β-sheets that are formed by residues 18–26 and 31–42 [6]. Thereafter, another model for Aβ amyloid with a similar motif was proposed, known as the Ma-Nussinov-Tycko model [10]. The strand-bend-strand structures were also resolved in Aβ_1–40_ fibrils with β-strand secondary structure in residues 11–22 and 30–39, based on numerous constraints from solid-state NMR and electron microscopy (EM) [11]. In 2013, a 3-fold structure of brain-derived Aβ_1–40_ fibrils was detected by Tycko group through solid-state NMR [12]. The fibrils from two AD patients display a distinct morphology. The determined molecular model includes a twist in residues 19–23, a kink at G33, and a bend in glycine residues 37 and 38. Recently, a structure of an Aβ_1–42_ fibril composed of two twisted protofilaments was resolved by cryoelectron microscopy (cryo-EM) [13]. The atomic model is comprised of β-strands in residues 1–9, 11–20, and 27–33, with a kink around Tyr10 and a turn region of residues 21–25. Overall, Aβ_1–40_ and Aβ_1–42_ fibrils show a high structural polymorphism.

Two key regions, the central residues 16–22 (β1) and the C-terminal residues 30–36 (β2), favor β-sheet formation and promote the assembly of Aβ to form higher-order oligomers, which can also coassemble antiparallel to form β-hairpin-rich oligomers [14–16]. Shorter Aβ fragments are also observed to aggregate in brain tissue [17], and it provides an ideal model to characterize Aβ oligomerizaion and pathology. Thus, truncated Aβ fragments have been synthesized and studied, such as Aβ_16–22_ [18], Aβ_10–35_ [19] and Aβ_25–35_ [20] peptides. The fragment of Aβ_16–22_, comprising β1-strand of the fiber models, has been extensively investigated by experiments and computational simulations. NMR study by Balbach et al. showed that the fragment of Aβ_16–22_ could form highly ordered fibrils with an antiparallel organization of β-sheets, similar to those by full-length Aβ [21]. Spectroscopic study by Lu et al. reported that the termini-capped Aβ_16–22_ peptides assembled into fibers and nanotubes at neutral pH [22]. Performing atomistic replica exchange molecular dynamics (REMD) simulations, Gnanakaran et al. reported that Aβ_16–22_ could form stable dimers aligned in parallel, antiparallel, or cross patterns [23]. Using all-atom MD simulations with explicit solvent, Nguyen et al. monitored the growth of Aβ_16–22_ oligomer by adding a disordered monomer to an ordered oligomer, and they found the dynamics of oligomer formation follows a two phase dock-lock mechanism [24]. These studies have provided important information on the structural and biophysical information of small Aβ_16–22_ oligomers. Nevertheless, the knowing of the Aβ_30–36_ fragment that comprises β2-strand, remains elusive.

The microcrystal structure of Aβ_30–35_ segment was identified by Colletier et al. through its X-ray fiber-diffraction pattern [25]. They found the fibrillar structure displays one type of parallel β-sheet with a face-to-back steric zipper, which forms a knobs-into-holes type of packing. Thereafter, a β-sheet amyloid mimic (BAM) derived from Aβ_30–36_ was crystallized [26], and recognized to form out-of-register fibrils or cylindrin-like, out-of-register oligomers, which bond face to face [27]. Both types of BAM (Aβ_30–36_) are toxic to mammalian cells, whereas the in-register peptide fibrils formed by Aβ_30–36_ show little toxicity. The contrast of peptide interfaces reflects the structural polymorphism of Aβ_30–36_ oligomers, and needs to be further clarified. The unnatural amino acid Hao applied in BAM (Aβ_30–36_) blocks uncontrolled intermolecular hydrogen-bonding and promotes β-sheet formation. Therefore, the pure Aβ_30–36_ oligomers are supposed to have a more complex hydrogen-bonding network with respect to BAM (Aβ_30–36_); meanwhile, the dominant interaction of Aβ_30–36_ peptide assembling also needs further investigating. Here we carried out replica-exchange molecular dynamics (REMD) simulations in explicit solvent to characterize the atomic structure of capped Aβ_30–36_ hexamers and examine the key driving force for the oligomerization of Aβ_30–36_ peptides. We also studied two mutations of Aβ_30–36_ (G33V and L34T) implicated in the stability of the wild type (WT), and compared their assembling behaviors with the WT peptides. It might provide a potential explanation of the reduced neurotoxicity induced by G33V/L34T Aβ peptides.

## Materials and methods

### Peptide models

Six Aβ_30–36_ (30AIIGLMV36) peptides were placed randomly in a simulation box (5.6nm×5.6nm×5.6nm), and then underwent a 5-ns simulation at 500 K to make the residues in the state of coil. The peptides are at neural pH with the N- and C-terminus in capped state, and counterions are added to neutralize the system and mimic the experimental condition. We choose Aβ_30–36_ hexamer for two reasons. First, Liu et al. found that β-sheet amyloid mimic (BAM) Aβ_30–36_ may form cylindrin-like tetramer using X-ray crystallography [27]. The hydrogen bond network of BAM(Aβ_30–36_) is similar to that of two-stranded antiparallel β-sheets, while it has a strong interface and a weak interface. Since the pure Aβ_30–36_ can provide more hydrogen bonding sites, the peptide number essential to form Aβ_30–36_ β-barrel should be close to and less than eight. Second, previous Monte Carlo [28] and REMD [29] studies showed that a β-barrel can be formed by 6~8 Aβ_16–22_ peptides. As Aβ_30–36_ peptide has the same amino acid length as Aβ_16–22_, it probably needs no more than eight chains to form a stable closed β-barrel. Since the hexamer system needs the least computational resource, we adopt Aβ_30–36_ hexamer for REMD simulations.

Several mutations located at the Aβ_30–36_ fragment have been studied, such as Piedmont mutant L34V, Iowa mutation G33N, etc. The L34V mutant shows a similar hemorrhagic phenotype and elicits an analogous cell-death pathway as the WT Aβ_1–40_ peptide [30]. It also leads to the aggregation and deposition of Aβ_1–42_ in the brain and causes the Piedmont type of hereditary cerebral amyloid angiopathy [31]. Among these sing-point substitutions, two mutations G33V and L34T attract our attention, because they were reported to reduce Aβ toxicity [32–36]. Kanski et al. investigated the effect of G33V on Aβ_1–42_-induced oxidative stress and neurotoxicity in cultured hippocampal neuronal cells, which shows that G33V mutant peptides only oxidize neuronal proteins to a small extent, and cause no significant cell death [33]. Lecomte group studied the structures of Aβ_1–42_ and its L34T mutant, and found the mutants are harmless, which may be attributed to incapacity for L34T mutants to form anti-parallel β-sheet fibrils [34]. The systems of G33V mutant (ACE-AIIVLMV-NH_2_) and L34T (ACE-AIIGTMV-NH_2_) mutant peptides were pre-simulated at 500 K for 5 ns as the WT system did. The obtained structures were used to as the initial states for REMD simulations (shown in S1 Fig).

### REMD simulations in explicit water

Atomistic MD simulations are performed in the isothermal-isobaric (NPT) ensemble using the GROMACS 4.5.3 software [37]. GROMOS96 force field [38] has been widely applied to model Aβ peptides in plenty of computational studies [24,39–42], and it can obtain Aβ conformational propensities in agreement with NMR results [43]. Previous MD/REMD simulations show that GROMOS96 force field is suitable for studying the Aβ aggregation, as well as the interaction between Aβ and nanoparticles, small molecules, etc. Therefore, we select GROMOS96 force field to model Aβ_30–36_ fragments. The water molecules are modeled by SPC [44]. We perform three REMD simulations [45–47] to study Aβ_30–36_ hexamer and its G33V/L34T mutants, with a total simulation time of 18 μs. Each system includes 40 replicas, and one replica is a single 150-ns MD run at specific temperature ranging from 305 K to 430 K. The time step used in MD simulations is 2 fs, and the replica exchange is attempted every 1000 steps, resulting in an average acceptance ratio of ~22% for each system. Peptide bonds are constrained by the LINCS algorithm [48] and water geometries are constrained by SETTLE [49]. The temperature is maintained constant using velocity rescaling method [50], and the pressure is kept at 1 bar using the isotropic Parrinello-Rahman’s method [51,52]. Long-range electrostatic interaction is calculated using the PME method [53] with a real space cutoff of 1.0 nm, and van der Waals interaction is calculated using a cutoff of 1.4 nm.

### Analysis methods

The Daura analysis method [54] was applied to cluster the sampled conformations using a Cα-root mean square deviation (Cα-RMSD) cutoff of 0.35 nm. A hydrogen bond (H-bond) is considered to be formed if the distance between donor D and acceptor A is less than 3.5 Å and the D-H-A angle is larger than 150°. A β-sheet angle is defined as the angle between two neighboring β-strands in all size of β-sheets. The twist angle of a β-sheet bilayer is averaged over all the involved β-sheet angles (supplementary angle if the β-sheet angle is obtuse). The DSSP algorithm [55] is applied to determine the secondary structure. Based on the interlayer topology, the conformations of bilayer β-sheets are denoted by m + n, where m and n respectively represent the m- and n-stranded β-sheets forming the bilayer. More simulation details are given in the Supporting Information.

## Results and discussion

We verified the convergence of the three REMD simulations through the comparison of the following parameters within two different time interval using 50–100 and 100–150 ns data for WT, G33V and L34T systems, respectively: (1) probability density function (PDF) of end-to-end distance for all peptides, number of H-bonds, radius of gyration (RG), and solvent accessible surface area (SASA); (2) the average population of different secondary structure contents (Supporting information). As shown in S2 Fig, the distributions of the reaction coordinates mentioned above within two independent time intervals overlap very well for all the systems. The secondary structure contents for three systems are also quite similar within two independent time intervals, shown in S3 Fig. These results suggest that our REMD simulations are reasonably converged.

### WT, G33V and L34T Aβ_30–36_ peptides in the oligomers display differences in secondary and tertiary structures

**Fig 1:**
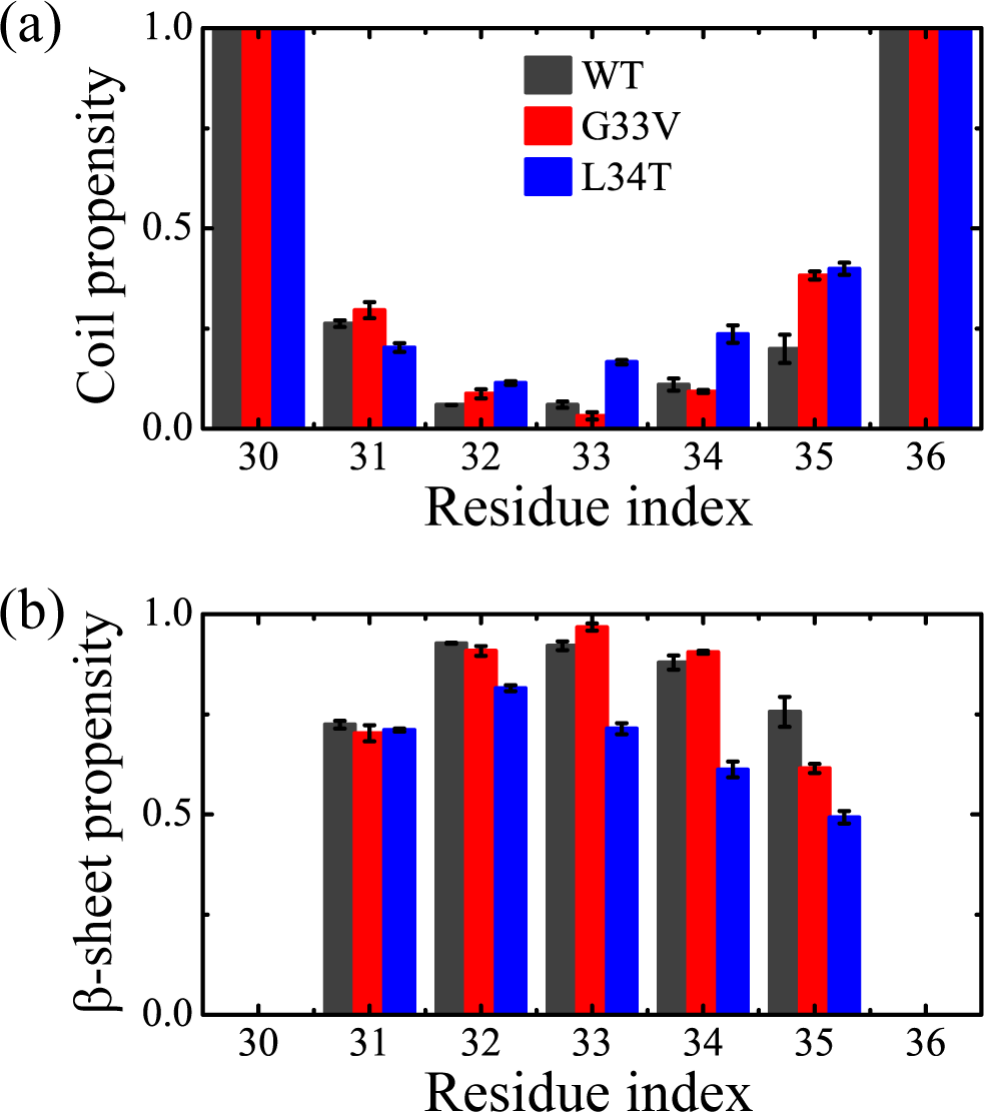
**Propensity of dominant secondary structure contents for each amino acid residue in wild type (WT), G33V and L34T Aβ**_**30–36**_ **hexamers at 310K: coil propensity (a) and β-sheet propensity (b).** Error bars were calculated using the 50–100 ns and 100–150 ns data.

To examine the structural properties of WT, G33V and L34T aggregates at 310K, we calculated the dominant secondary structure (coil and β-sheet) probability of each residue in Fig 1. It shows that Aβ_30–36_ hexamer has an average β-sheet content of 60.1% in all at 310 K, and the residues located at the middle of the WT Aβ_30–36_ sequence have a higher probability to adopt β-sheet conformations than those at the ends. The inclination of Aβ_30–36_ oligomers for β-sheet formation is in good agreement with experimental observations [15,20,27,56]. After the substitution of G33 by V, the average coil propensity is slightly increased (from 38.4% to 41.3%) and the β-sheet propensity reduced, in which M35 contributes the most. Interestingly, previous studies suggest that G33 is a possible site of free radical propagation processes that are initiated on M35, which is involved in Aβ toxicity [32,33]. Although our model cannot simulate the proton transfer process, the results still reflect that G33V mutation influences the structure of M35 the most among all the residues. Meanwhile, nearly all the residues of the L34T mutants show an increment in coil propensity and a reduction in β-sheet propensity. The difference of secondary structures between the WT peptides and mutants reflects that the substitution of L34 by T has a significant prevention of the β-sheet formation of Aβ_30–36_ hexamer. Recent spectroscopic studies show that the L34T mutation alters the oligomeric structure and prolongs the lag phase of Aβ_1–42_ fibrillation, accompanied with decreased toxicity [34–36]. The β-sheet reduction of Aβ_30–36_ segments duo to the L34T substitution, is supposed to go against the fiber formation of full-length Aβ_1–42_ and the oligomerization at the early stage. These results indicate that the WT Aβ_30–36_ hexamer prefers to form β-sheet, and the G33V and L34T mutations have a different influence on the secondary structure of Aβ_30–36_ assemblies.

**Fig 2:**
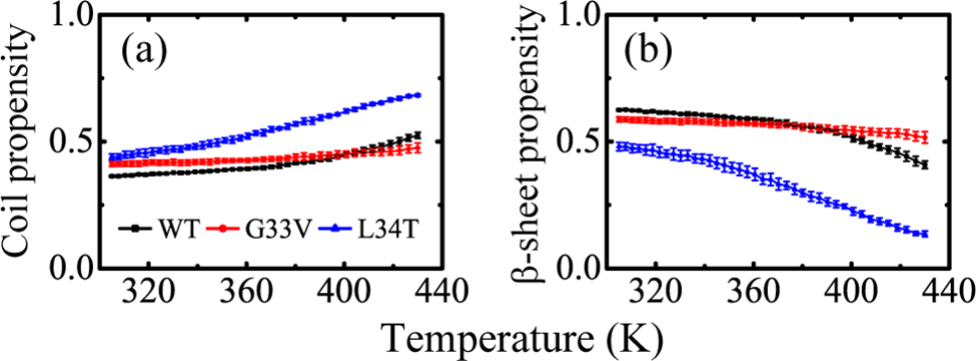
**Average coil (a) and β-sheet (b) propensities of WT, G33V and L34T Aβ**_**30–36**_ **hexamers at different temperatures.** The error bars were calculated using the 50–70 ns, 70–90 ns, 90–110 ns, 110–130 ns, and 130–150 ns data.

The temperature dependence of secondary structures for WT, G33V and L34T Aβ_30–36_ hexamers was examined by calculating their average coil and β-sheet propensities, shown in Fig 2. As the temperature increases, the WT Aβ_30–36_ hexamers display a gradual rising coil propensity, with 36.4±0.3% at the lowest temperature of 305 K and 52.5±1.2% at the highest temperature of 430 K, and a gradual decline of β-sheet propensity, with 62.5±0.3% at 305 K and 40.9±1.5% at 430 K. This indicates that the WT Aβ_30–36_ peptides prefer to aggregate at lower temperatures, consistent with other amyloid sequences, such as p53 aggregation-nucleating ^251^ILTIITL^257^ [57], Aβ_16–22_ [29] and Aβ_29–42_ peptides [58]. As for G33V mutants, they have similar temperature dependence as WT, with a β-sheet propensity of 58.8±1.0% at 305 K and 51.6±2.3% at 430 K. With respect to WT hexamers, the G33V mutants have a lower β-sheet propensity in the range of 305–383 K and a higher β-sheet propensity in the range of 387–430 K. As for L34T mutants, they have a significantly lower β-sheet propensity than WT peptides at all the simulated temperatures, with a propensity of 48.0±1.6% at 305 K and 13.6±1.1% at 430 K.

**Fig 3:**
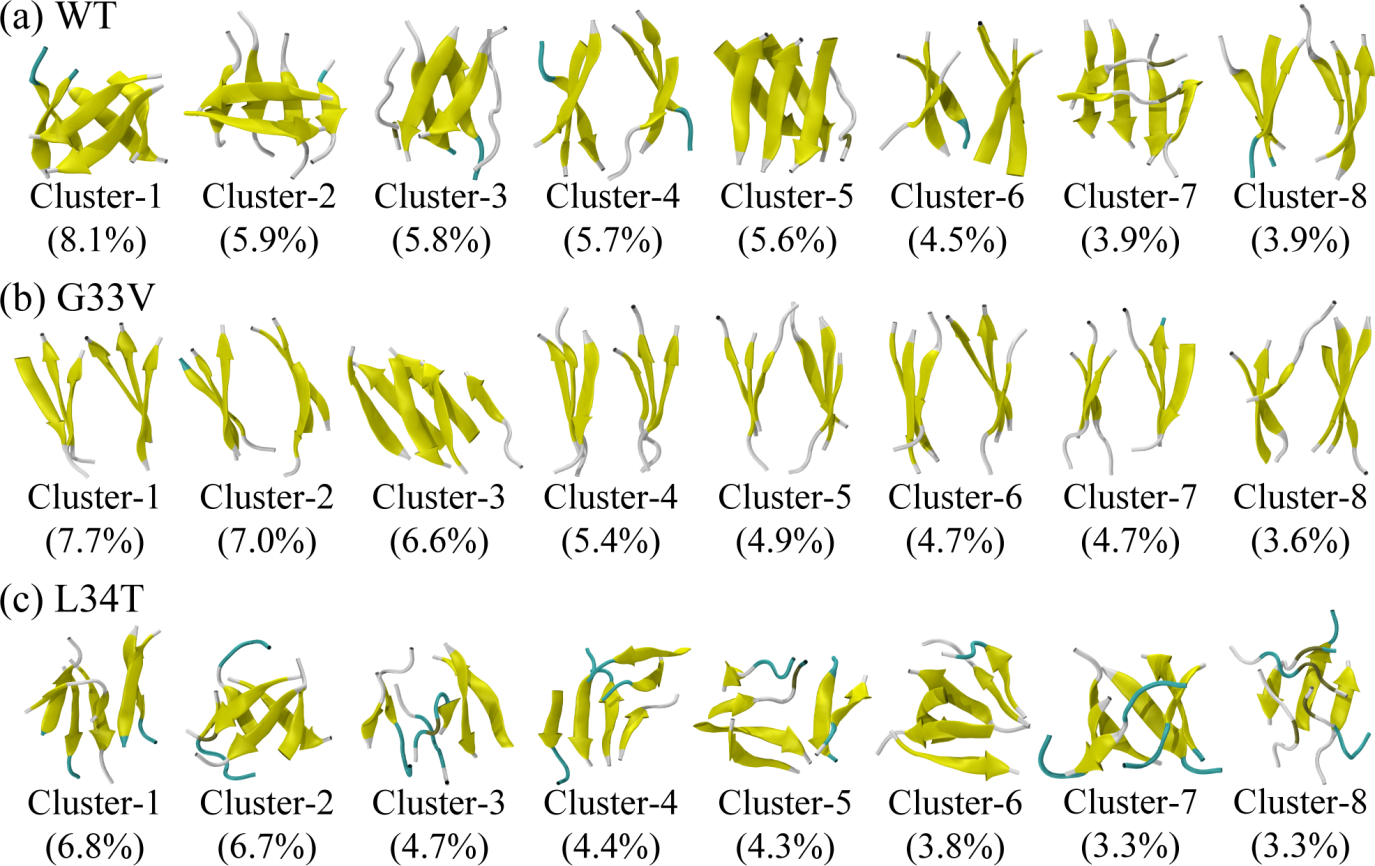
**Representative conformations of the first eight most-populated clusters for WT (a), G33V mutant (b), and L34T mutant (c) Aβ**_**30–36**_ **hexamers at 310K.** The corresponding population of each cluster is given in the parentheses.

We then performed a RMSD-based cluster analysis for 50000 conformations sampled for each system at 310K using the procedure described in the methods subsection. Using a Cα-RMSD cutoff of 0.35 nm, the conformations of Aβ_30–36_ WT and its G33V/L34T mutant oligomers are separated into 139, 112 and 266 clusters, respectively. Fig 3 shows the central structure and corresponding population of the first eight most-populated clusters. These clusters represent 43.4%, 44.6%, and 37.3% of all conformations of wild type, G33V and L34T hexamers, respectively. The Aβ_30–36_ WT hexamers primarily adopt ordered β-sheet-rich conformations, comprised of β-barrel, 4 + 2 and 3 + 3 β-sheet bilayer, in good agreement with the X-ray study in which the crystallographically determined BAM Aβ_30–36_ can form cytotoxic β-barrel-like oligomers and fibrils [27]. A closed β-barrel hexamer was observed in Cluster-1, with the β-strands in out-of-register alignments. Conventional MD simulations initiated from Cluster-1 display a small RMSD (<0.2 nm) as a function of time (see S8 Fig), revealing that the β-barrel structure is quite stable. Similar β-barrels composed of Aβ_16–22_, Aβ_25–35_ or K11V display a stable existence in the previous computational or experimental studies [20,28,59,60], suggesting that the cylindrin-like oligomer is probably an on-pathway intermediate involved in out-of-register amyloid fibril formation [27]. These cylindrins and β-barrels can reduce cell viability via a proposed mechanism of interacting with membranes [61–63]. Through conventional MD simulations, the 4 + 2 β-sheet bilayer is observed to be able to transfer to a closed barrel-like structure (see S4 Fig), accompanied with enlarged structural fluctuation and a β-sheet-coil transformation when the H-bonds are destructed. The β-strands in one bilayer are orthogonal to those in the other bilayer at first, and the two-stranded bilayer can drift away from the four-stranded bilayer. The structural flexibility of the 4 + 2 β-sheet bilayer in Cluster-2 implies that it is probably an intermediate in touch with β-barrel and 3 + 3 β-sheet bilayer. Similar structural flexibility of fibril-barrel transitions has recently been observed by Zhang et al. in a replica-exchange-with-tunneling simulation study of three K11V peptides [64]. They found a key transition state connecting the fibril and cylindrin pathways, in which peptides have not yet associated by inter-chain H-bonds.

Compared with the conformations of Aβ_30–36_ WT oligomers, those of G33V mutant have a higher similarity and predominantly adopt β-sheet-rich bilayers. Among the first eight most-populated clusters, 4 + 2 β-sheet bilayer is observed in Cluster-3, and 3 + 3 β-sheet-rich bilayers in all the other clusters. We also observed well-formed β-sheet twists in the 3 + 3 β-sheet bilayers, whose twist angles range from 12 to 32 degree. Our result is larger than the twist angle obtained from the well-formed fiber structure [18,65–67]; because the simulated hexamer has a much smaller size than the mature fiber, and it is easily interfered by water molecules. The inter-mainchain H-bonding map of the first eight most-populated clusters in S5 Fig shows that the G33V Aβ_30–36_ peptides can adopt in-register parallel (major) or antiparallel (minor) β-sheets, and two-residue-shift out-of-register parallel (medium) β-sheets, evolutionarily more optimized by β-sheet twists. Similar twisted morphologies of antiparallel β-sheet have been reported in the study of membrane-bound Aβ pore and β2m_83–89_ oligomers [68,69]. On the contrary, the L34T mutants display a higher diversity and have more β-bridge (tan-colored parts in Clusters-2, 3, 5 and 8) as well as bend (cyan-colored parts) content. The disordered structures are hard to classify according to their tertiary topology, and small hairpin loop and hairpin are observed in Clusters-1 and 3, respectively. The ACE group, residues I31, T34 and V36 of one peptide in Cluster-1 is observed to form H-bonds with the closest peptides, as shown in S6 Fig. This hairpin loop peptide helps to stabilize the oligomeric structure through H-bonding with the neighbor peptides. One peptide in Cluster-3 displays a hairpin conformation with two pairs of intrachain H-bonds (I32-NH2 and I32-M35, shown in S6 Fig). This hairpin peptide also forms H-bonds with intralayer strand, but does not form H-bonds with interlayer peptide.

**Fig 4:**
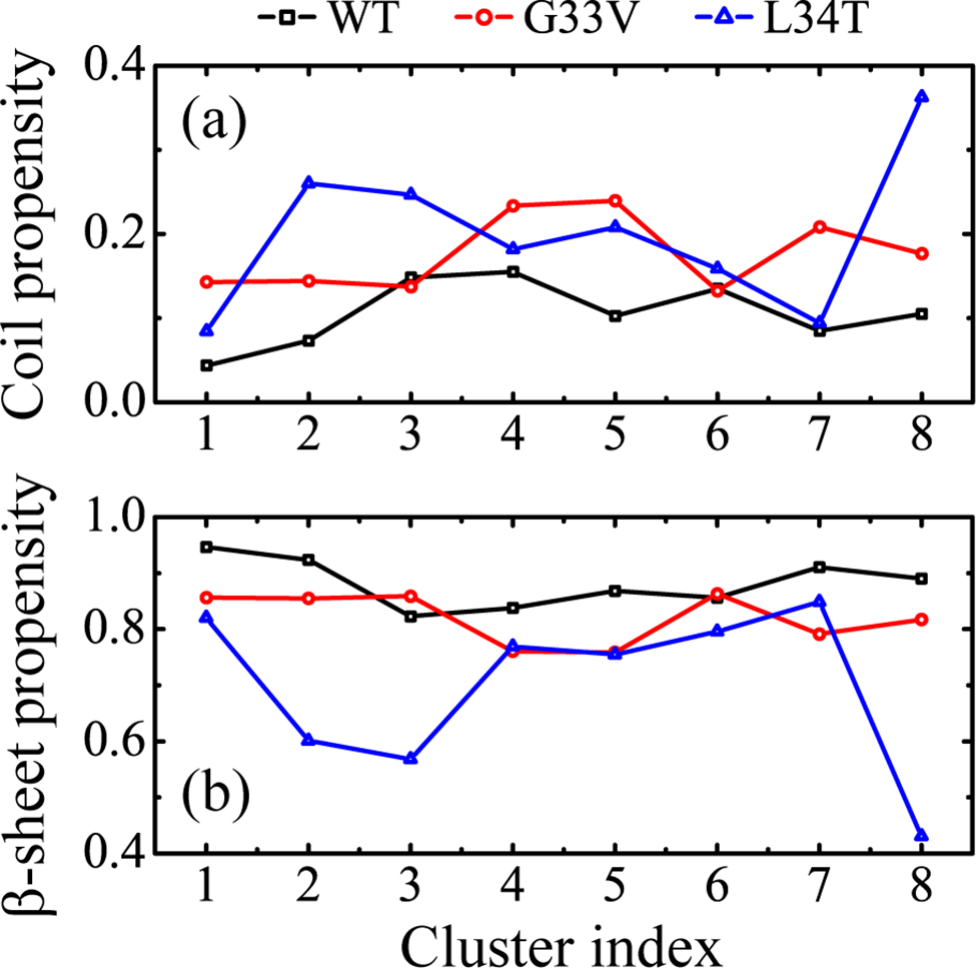
**Averaged propensity of coil (a) and β-sheet (b) as a function of cluster index in WT, G33V and L34T Aβ_30–36_ hexamers at 310K**.

In order to investigate the dominant secondary structure (coil and β-sheet) probability of the most-populated conformations, we calculated the coil and β-sheet propensity as a function of cluster index for each system at 310K in Fig 4. It shows that the WT and G33V/L34T Aβ_30–36_ hexamers display a different secondary structure content distribution. Using Daura cluster analysis method, the cluster with lower index has a higher sampling probability. Although the substitution of G33 by V has a slight influence on the total population of secondary structure, it remarkably increases the coil component in the first eight most-populated conformations, despite of a slight decrease of 1.1% for Cluster-3 and 0.2% for Cluster-6; it also reduces the β-sheet component, despite of a slight increase of 3.6% for Cluster-3 and 0.7% for Cluster-6. This indicates G33V mutant has a lower probability to form β-sheet-rich structures with respect to WT Aβ_30–36_. The difference of WT and G33V mutant peptides in secondary structure may result from the change of backbone dihedral angle distribution, and the peptides still favor to form β-sheet (see S7 Fig). It is reminiscent of a previous computational study, in which Lu et al. applied the coarse-grained protein OPEP force field to investigate the effects of G33I mutation on the structures of Aβ_29–42_ monomer and dimer. Interestingly, they found the G33I mutants are more disordered than WT dimer, and display less β-sheet content in the C-terminal residues, with a slightly increased population of parallel alignments [58]. As for the substitution of L34 by T, it not only reduces the β-sheet content of Aβ_30–36_ hexamer, but also significantly reduces the probability to form β-sheet-rich structures. These indicate that both G33V and L34T mutant Aβ_30–36_ hexamers have lower β-sheet content than WT peptides in the most-populated conformations.

### WT Aβ_30–36_ peptides mainly assemble into β-barrel or β-sheet-rich oligomers, while G33V substitution leads to more extended β-sheet-rich hexamers, and the L34T mutants have more complex H-bonding network

**Fig 5:**
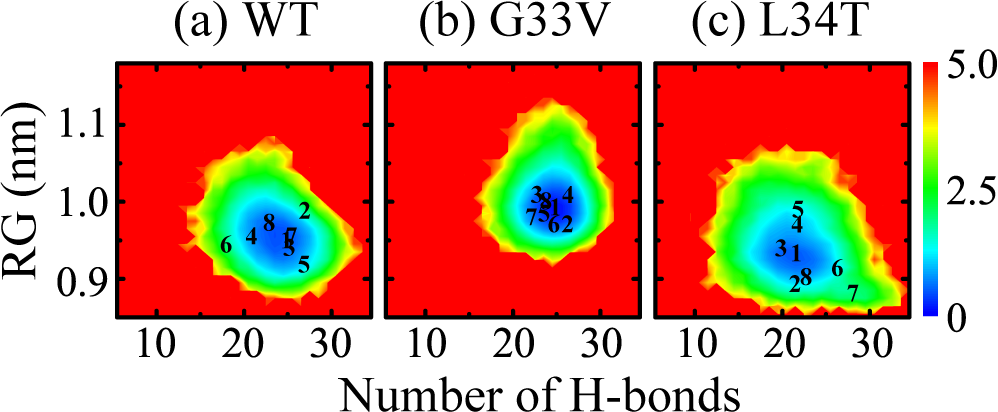
**Free energy landscape (in kcal/mol) for WT (a), G33V mutant (b), and L34T mutant (c) Aβ**_**30–36**_ **hexamers at 310 K, projected in the two-dimensional plane of the intermolecular H-bond number and radius of gyration (RG).** Cluster numbers corresponding to the representative structures of three systems in Fig 3 are respectively marked in the plane.

To have an overall view of the conformational distribution of WT and G33V/L34T mutant Aβ_30–36_ oligomers, we plotted the two-dimensional free energy landscape (or potential of mean force, PMF) as a function of H-bond number and RG in Fig 5. As shown in Fig 5A, the free energy surface of WT Aβ_30–36_ oligomers is broad and the minimum-energy basin is shallow, with the number of H-bonds ranging from 12 to 35 and the RG ranging from 0.85 to 1.1 nm. Considering the centers of the most-populated conformations (including β-barrel, 4 + 2 and 3 + 3 β-sheet bilayer) projected in this H-bond number-RG plane are close, twenty-four 100-ns independent conventional MD simulations were performed to check if these structures can convert to each other. The low converting ratio (one out of 24 runs, see S8 Fig) indicates that the transformation is energetically very costly, suggesting that the oligomeric structures might be involved in distinct aggregation pathways of Aβ_30–36_ peptides. Especially for the intralayer rearrangement of strand alignment, a complete H-bonding network needs reforming.

After the substitution of G33 by V, the intermolecular H-bond number of Aβ_30–36_ hexamers ranges from 15 to 33, and the RG ranges from 0.90 to 1.15 nm. The increased RG reflects that the β-barrel structures are distinctly reduced and more residues are exposed to water solution. The hydrophobicity of valine makes the peptides less compacted, which implies the geometric occupation of its sidechain disturbs the packing of residue sidechain and as a result changes the oligomeric morphology. As for the L34T Aβ_30–36_ oligomers, the H-bond number ranges from 9 to 35, and the RG ranges from 0.85 to 1.08 nm. The more disperse distribution of H-bond number indicates that the L34T hexamers have reduced β-sheet content and more disordered structures.

**Fig 6:**
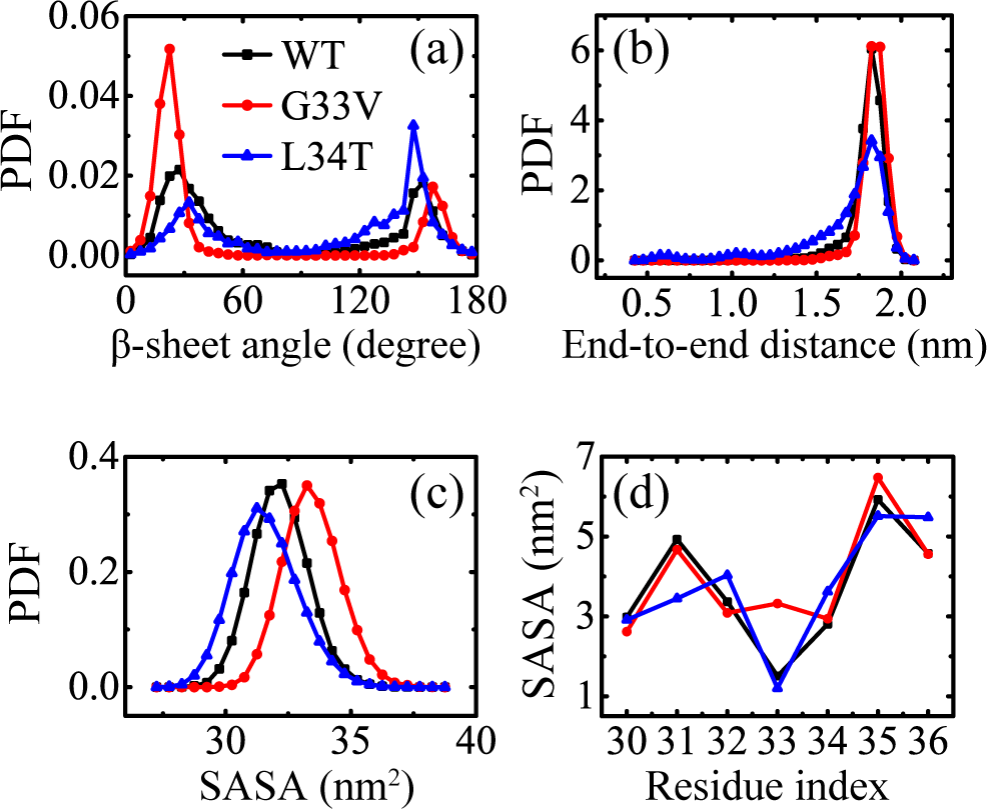
**Analyses of peptide β-sheet angle, end-to-end distance, and solvent accessible surface area (SASA) for WT, G33V mutant, and L34T mutant Aβ_30–36_ hexamers at 310 K:** (a) probability density function (PDF) of β-sheet angle; (b) PDF of end-to-end distance for all peptides; (c) PDF of SASA for all residues; (d) SASA as a function of residue index.

Distributions of β-sheet angle of WT and G33V/L34T mutant Aβ_30–36_ oligomers are given in Fig 6A to quantify the parallel and anti-parallel β-sheet frequency. It shows that the β-sheets formed by WT Aβ_30–36_ peptides have a slight preference for parallel orientation, with a parallel/anti-parallel percentage of 61.6%/38.4%; G33V β-sheets significantly tend to be parallel aligned; L34T β-sheets have a preference of out-register antiparallel alignment. The end-to-end distance probability distributions for all chains are presented in Fig 6B. There is a sharp peak located at 1.8 nm in WT and G33V peptide systems, corresponding to β-strand or β-sheet-rich conformations. The structural ensemble of L34T peptides gets broader, with the highest peak located at 1.8 nm and two smaller peaks at 0.6 nm and 1.1 nm (see S2A Fig for clarity), respectively. The PDF peak of 0.6 nm for a L34T mutant corresponds to the hairpin structure, and the peak of 1.1 nm corresponds to the meander structure, namely hairpin loop (shown in S6 Fig). These indicate that L34T peptide in the oligomers is less extended than WT peptide, corresponding to less β-sheet content. Fig 6C and 6D show the probability distribution of SASA and the contribution to SASA per residue, respectively. The G33V mutant oligomers display an increased surface area exposed to water than the WT Aβ_30–36_, attributed to the hydrophobic sidechain of valine. As for the L34T mutant oligomers, the SASA of A30, I32, L34 and V36 is increased, while the SASA of I31, G33 and M35 is reduced. The adjacent switch of SASA increment reveals that the I31-G33-M35 face of the Aβ_30–36_ peptide prefers to orientate to the interior of oligomers after the L34T substitution. Note that if the SASA is normalized by the total surface area of all chains (see S9 Fig), the enhanced hydrophobicity induced by the G33V mutation indeed decreases the normalized SASA, and the distribution of normalized SASA for L34T mutant oligomers remain the same as the WT peptides.

### WT and G33V oligomers are mostly stabilized by hydrophobic interaction, while L34T oligomers are stabilized by both hydrophobic and hydrogen-bonding interactions

**Fig 7:**
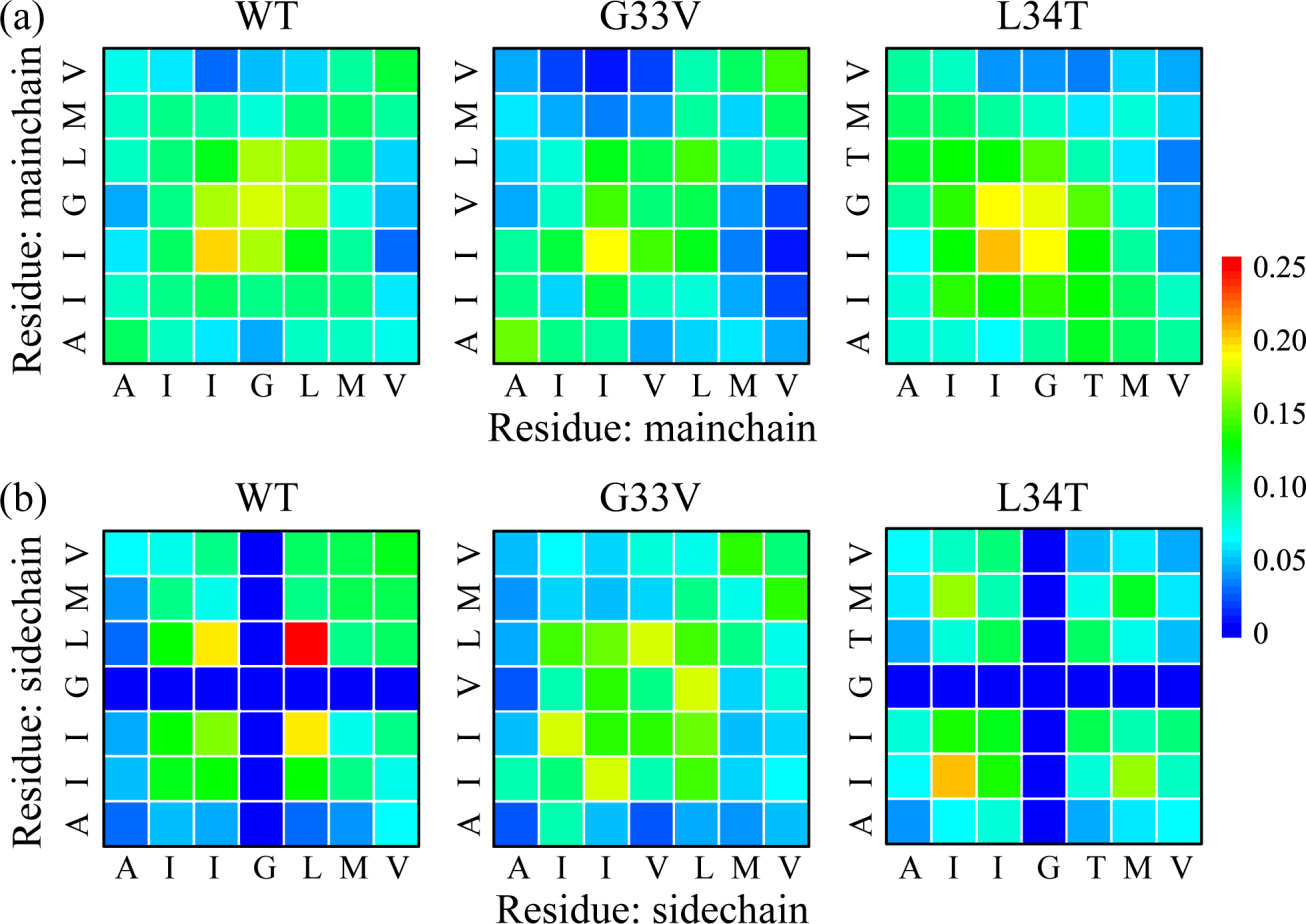
**The interpeptide mainchain-mainchain (MC-MC) (a) and sidechain-sidechain (SC-SC) (b) contact probability maps of REMD-generated conformations of WT, G33V mutant, and L34T mutant Aβ_30–36_ oligomer at 310 K**.

To probe the primary peptide-peptide interactions disturbed by the mutations and the key residues for β-sheet formation, we plotted in Fig 7 the mainchain-mainchain (MC-MC) and sidechain-sidechain (SC-SC) contact probability map between all residue pairs of WT, G33V mutant, and L34T mutant Aβ_30–36_ oligomer, respectively. The relatively smooth MC-MC contact probabilities of WT peptides reveal that the capped Aβ_30–36_ peptides assemble with no preferred orientation. The I32-I32, I32-L34 and L34-L34 pairs have a high SC-SC contact probability of 15.8%, 19.4%, and 26.5%, respectively, indicating the hydrophobic interaction plays an important role in peptide-peptide interplay. This is consistent with a recent NMR study of macrocyclic Aβ_30–36_ tetramer, in model of which the sidechains of I32 and L34 have a strong packing [56].

When G33 is substituted by V, the peptides present weakened MC-MC contacts and have a tendency to be parallel aligned. The SC-SC contacts show the interaction between the sidechains of I32 and L34 are greatly lessened, and those of I31-I32 and V33-L34 pairs are enhanced. It reveals that the increased hydrophobicity brought by G33V mutation alters the associations between other residues and also interferes with the MC-MC interaction. Too much hydrophobic interaction is reported to have a negative effect on the protein stability as well as the formation of an aggregative nucleus in peptides-hydrophobic surface system [70], or to reduce the β-sheet content of fibrils and lead to disordered oligomers in Aβ_16–22_-crowder system [71]. In a previous REMD simulation study of G33I mutant Aβ_29–42_ dimer, the enhanced hydrophobic at G33 was also reported to reduce intermolecular interaction in WT dimer [58]. Our REMD results show that the hydrophobic sidechain of V33 disrupts the β-barrel and increase coil content in most-populated clusters of Aβ_30–36_ assemblies. After the substitution of L34 by T, the MC-MC contact map displays a much higher probability along the left diagonal, indicating that the L34T mutant has a preference of out-register antiparallel alignment and the C-terminal residues tend to be oriented to water environment. This agrees with the structural characterization of less toxic Aβ mutant (L34T) fibrils using tip-enhanced Raman spectroscopy, in which the mutants align slightly less parallel and more antiparallel compared with WT peptides [36]. For SC-SC interactions, the interplays between I31 and M35 (I31-I31, I31-M35, and M35-M35 pairs) have the highest contact probabilities in replace of those between I32 and L34, indicating the L34T substitution makes the sidechain of I31 and M35 prefer to be buried in the interior of oligomers, which is consistent with the SASA analyses in Fig 6. These are attributed to the hydrogen bonding between T34 and other chains and the increased hydrophilicity of C-terminal residues brought by L34T mutation.

**Fig 8:**
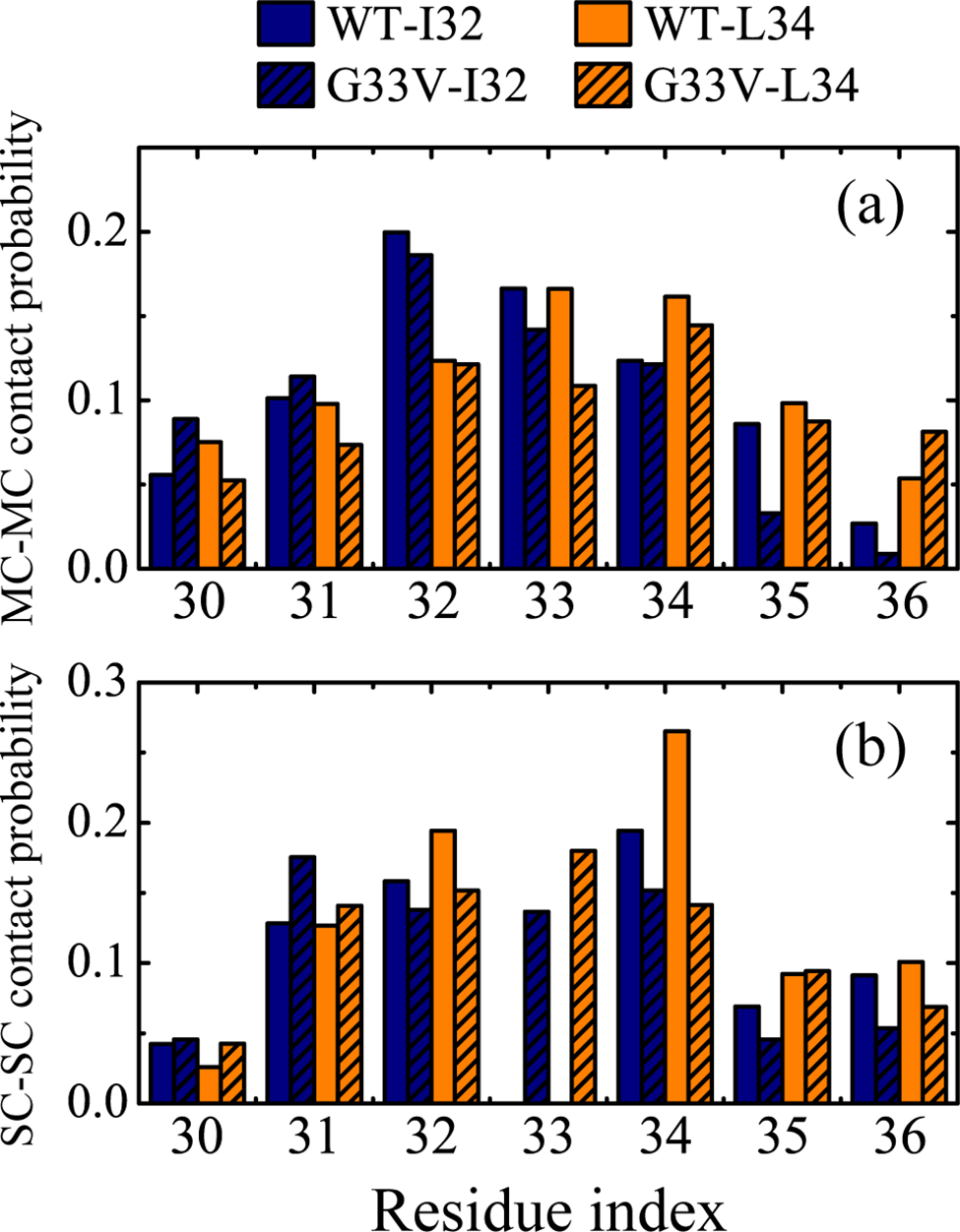
**The interpeptide MC-MC (a) and SC-SC (b) contact probability between I32/L34 and other residues for WT and G33V system at 310 K.** The blue and orange columns correspond to I32 and L34, respectively; the blank and filled columns correspond to WT and G33V Aβ_30–36_ oligomers, respectively.

To clarify the difference of peptide interaction as a result of the G33V substitution, we presented the interpeptide MC-MC and SC-SC contact probability between I32/L34 and other residues in Fig 8. The MC-MC contacts become less in all; the SC-SC contact probabilities of I32 and L34 get smaller after the G33V substitution, whereas those of I31 and M35 become higher (except for a decrease of 2.3% for I32-M35 pair that may be involved in other sidechain packing). According to the X-ray crystallographic observation [27], the cylindrin-like or fibrillar BAM (Aβ_30–36_) oligomer prefers to bond face to face, with the sidechains of I32 and L34 buried in interior, which is consistent with the highest SC-SC contact probability of I32 and L34 for WT peptides. After the substitution of G33 by V, the I31-V33-M35 face of peptide has an increment of hydrophobicity, and it competes with the I32-L34 face on sidechain packing. The steric-zipper effect between the I31-V33-M35 and I32-L34 face also makes the peptides tend to bond face to back. G33I is another mutation identified in Aβ_30–36_ region, with a similar physical and chemical nature. Harmeier et al. reported that this mutation promotes the aggregation process of Aβ_1–42_ by forming a continuous hydrophobic surface, and makes peptides more easily to form higher oligomers, which leads to less toxicity [72]. Reminiscently, the G33V substitution of Aβ_30–36_ peptides in our simulations makes the I31-V33-M35 face a continuous hydrophobic surface, which competes with the I32-L34 face in sidechain packing. As a result, the dry interior formed by I32 and L34 is disrupted, and the cylindrin-like Aβ_30–36_ oligomers are significantly reduced and the fibrillar oligomers increase.

**Fig 9:**
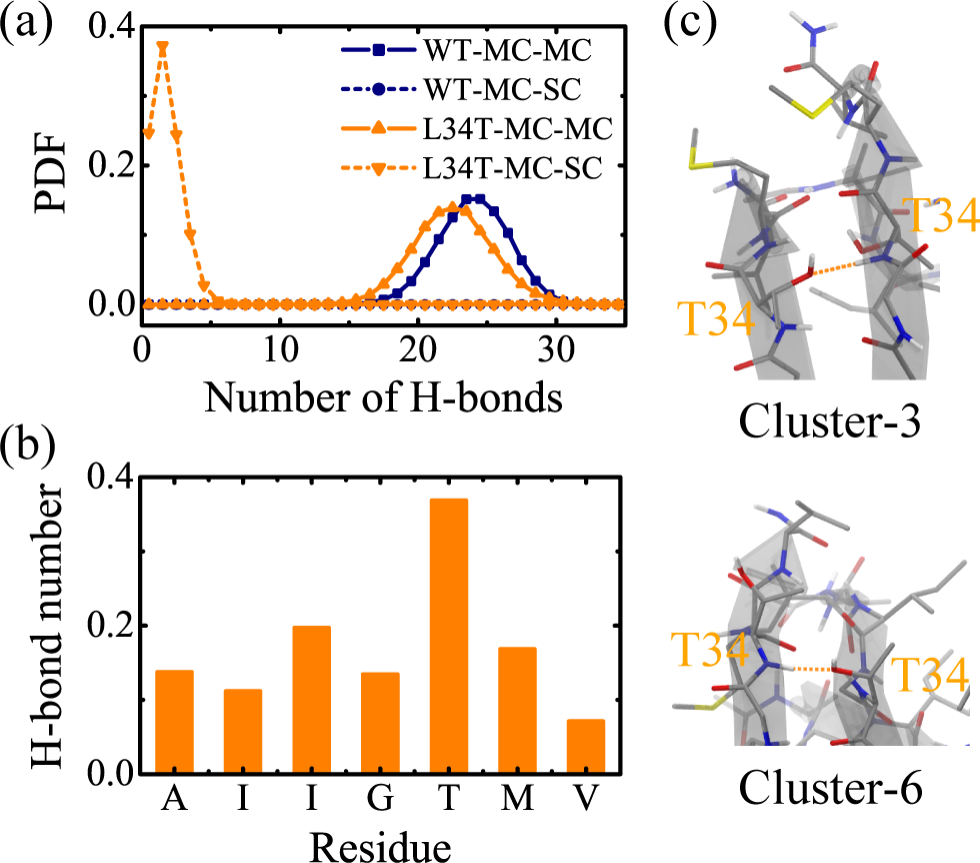
**Analyses of the intermolecular H-bond distribution for WT and L34T Aβ**_**30–36**_ **oligomer at 310 K: (a) the PDF of MC-MC and MC-SC H-bond number; (b) the average number of H-bonds formed between T34 sidechain and individual residue mainchain; (c) a local glance upon the conformations of Cluster-3 and Cluster-6.** The names of the residues involved in MC-SC H-bonding are highlighted in orange. The protein is represented in licorice, the secondary structure in cartoon, and the MC-SC H-bond in orange dashed line. Carbon atoms are colored in gray, oxygen atoms in red, nitrogen atoms in blue, sulfur atoms in yellow, and hydrogen atoms in white, respectively, in the snapshots.

We further examined the perturbation of the L34T substitution on the residue-based peptide-peptide interaction in the term of number of H-bonds, and plotted the intermolecular H-bond distribution in Fig 9A. It shows that the L34T oligomer form 1~2 less MC-MC hydrogen bonds and 1~2 more MC-SC hydrogen bonds than the WT peptides. The shift in the H-bond number is attributed to the formation of H-bonds between the hydroxyl groups of T34 sidechain and the backbone of Aβ_30–36_ peptides. As shown in Fig 9B, the sidechains of T34 favor the polar threonine with hydrogen bonding, and have no preference to the other hydrophobic residues. Fig 9C displays the snapshots of MC-SC H-bond formed in the conformations of Cluster-3 and Cluster-6, respectively. Our calculation and detailed structural check show that there is no sulfur atom involved in hydrogen bonding under the criterion mentioned in Methods section. It has been demonstrated that interpeptide hydrogen bonding and hydrophobic interaction play important roles in the formation and stabilization of Aβ aggregates [23,73–75]. Given the inability of WT Aβ_30–36_ sidechains to form H-bonds, the hydrogen bonding between sidechains of T34 and mainchains of other peptides disarranges the interpeptide H-bond network and leads to disordered oligomers.

## Conclusions

Using REMD simulations, we have investigated the hexameric structures of termini-capped Aβ_30–36_ peptides and examined the effect of G33V/L34T mutations on the oligomeric assemblies. Our results revealed that the Aβ_30–36_ hexamer has an average β-sheet content of 60.1% and mainly adopts conformations of β-barrels, 4 + 2 and 3 + 3 β-sheet bilayers at 310 K. The strands in β-barrels display out-of-register alignments; bilayer β-sheets include parallel, orthogonal, and antiparallel ones. The hydrophobic interaction between I32 and L34 residues plays a critical role in the assembly and structural stability of Aβ_30–36_ hexamers. The G33V mutants show less β-sheet contents in the most-populated (top 44.6%) conformations, and adopt β-sheet-rich bilayers on the whole, with strands mainly in in-register parallel alignments. The I31-V33-M35 face of peptides tends to orient to the I32-L34 face, and their steric-zipper effect interferes with the face-to-face sidechain packing of I32-L34 face in WT Aβ_30–36_. The L34T mutants have a significant β-sheet reduction and a higher structural diversity, including quite a few disordered and hairpin-like oligomers. It is mainly attributed to the hydrogen bonding of T34 sidechain with peptide backbones, which disturbs the intermolecular H-bond network in WT Aβ_30–36_. Overall, both the G33V and L34T mutations disrupt the Aβ_30–36_ β-barrel conformations that are closely related to cell toxicity, and weaken the I32-L34 hydrophobic interaction. Our REMD results provide structural insights into the assembly of WT and G33V/L34T mutant Aβ_30–36_ peptides, which is helpful for the development of amyloid-based nanostructures and the design of novel inhibitors.

## Supporting information

S1 Text Supplementary simulation details. (PDF)

S1 Fig **The initial structures of WT (a), G33V (b) and L34T (c) Aβ30–36 hexamer for REMD simulation.** The peptides are in cartoon representation.
(PDF)

S2 Fig **The probability density function (PDF) of end-to-end distance for all chains (a), number of H-bonds (b), radius of gyration (RG) (c), and solvent accessible surface area (SASA) (d) for three systems within two independent time intervals of 50–100 ns and 100–150 ns at 310K**.
(PDF)

S3 Fig Probability of secondary structures within two independent time intervals of 50–100 ns and 100–150 ns for WT, G33V and L34T Aβ_30–36_ hexamer systems at 310K. (PDF)

S4 Fig Cα-root mean square deviation (Cα-RMSD) of WT Aβ_30–36_ hexamer as a function of time, with corresponsive secondary and tertiary structures. Both conventional MD simulations are initiated from the 4 + 2 β-sheet bilayer in Cluster-2: (a) the peptides transfer to a closed barrel-like structure; (b) the two-stranded bilayer drifts away from the four-stranded bilayer.
(PDF)

S5 Fig The interpepide H-bonding map between backbones of the first eight most-populated conformations for WT, G33V and L34T Aβ_30–36_ hexamer systems. The color indicates the average number of H-bonds. AP0/P0 represents in-register antiparallel/parallel β-sheets; P1/P2 represents 1-residue-shift/2-residue-shift out-of-register parallel β-sheets.
(PDF)

S6 Fig **Snapshots of one L34T mutant Aβ**_**30–36**_ **peptide with an end-to-end distance of 5.77 Å (a), 9.66 Å (b) and 17.90 Å (c).** The pane-contained H-bonds are highlighted in orange, with explicit names of the residues involved in H-bonding. The end-to-end distance is calculated from the A30 Cα atom to the V36 Cα atom. The secondary structures are shown in cartoon representation, and the peptides in licorice representation with carbon atoms in cyan, oxygen atoms in red, nitrogen atoms in blue, sulfur atoms in yellow and hydrogen atoms in white.
(PDF)

S7 Fig **The distribution of dihedral angles of the first eight most-populated conformations for WT, G33V and L34T Aβ_30–36_ hexamers at 310K: (a) the probability in dihedral angle φ-ψ plane; (b) PDF of φ and ψ**.
(PDF)

S8 Fig The time evolution of Cα-RMSD for WT Aβ_30–36_ hexamer. These conventional MD simulations are initiated from the conformations in the first six most-populated clusters. Different colors represent independent MD runs. The green line of Cluster-2 corresponds to a transformation from a 4 + 2 β-sheet bilayer to a closed barrel-like structure.
(PDF)

S9 Fig The PDF of SASA which is normalized by the total surface area of all chains for WT, G33V and L34T Aβ_30–36_ hexamer systems at 310K. (PDF)

## References

[1] Alonso AdC, Zaidi T, Novak M, Grundke-Iqbal I, Iqbal K. Hyperphosphorylation induces self-assembly of τ into tangles of paired helical filaments/straight filaments. Proc Natl Acad Sci U S A. 2001; 98: 6923–6928. doi: 10.1073/pnas.121119298 11381127 10.1073/pnas.121119298 PMC34454

[2] Hardy J, Selkoe DJ. The amyloid hypothesis of Alzheimer's disease: Progress and problems on the road to therapeutics. Science. 2002; 297: 353–356. doi: 10.1126/science.1072994 12130773 10.1126/science.1072994

[3] Citron M. Strategies for disease modification in Alzheimer's disease. Nat Rev Neurosci. 2004; 5: 677–685. doi: 10.1038/nrn1495 15322526 10.1038/nrn1495

[4] Aguzzi A, O'Connor T. Protein aggregation diseases: pathogenicity and therapeutic perspectives. Nat Rev Drug Discov. 2010; 9: 237–248. doi: 10.1038/nrd3050 20190788 10.1038/nrd3050

[5] Petkova AT, Ishii Y, Balbach JJ, Antzutkin ON, Leapman RD, Delaglio F, et al A structural model for Alzheimer's β-amyloid fibrils based on experimental constraints from solid state NMR. Proc Natl Acad Sci U S A. 2002; 99: 16742–16747. doi: 10.1073/pnas.262663499 12481027 10.1073/pnas.262663499 PMC139214

[6] Lührs T, Ritter C, Adrian M, Riek-Loher D, Bohrmann B, Döbeli H, et al 3D structure of Alzheimer's amyloid-β(1–42) fibrils. Proc Natl Acad Sci U S A. 2005; 102: 17342–17347. doi: 10.1073/pnas.0506723102 16293696 10.1073/pnas.0506723102 PMC1297669

[7] Kayed R, Head E, Thompson JL, McIntire TM, Milton SC, Cotman CW, et al Common structure of soluble amyloid oligomers implies common mechanism of pathogenesis. Science. 2003; 300: 486–489. doi: 10.1126/science.1079469 12702875 10.1126/science.1079469

[8] Haass C, Selkoe DJ. Soluble protein oligomers in neurodegeneration: lessons from the Alzheimer's amyloid [beta]-peptide. Nat Rev Mol Cell Biol. 2007; 8: 101–112. doi: 10.1038/nrm2101 17245412 10.1038/nrm2101

[9] Selkoe DJ. Alzheimer's disease—genotypes, phenotype, and treatments. Science. 1997; 275: 630–631. 9019820 10.1126/science.275.5300.630

[10] Ma B, Nussinov R. Simulations as analytical tools to understand protein aggregation and predict amyloid conformation. Curr Opin Chem Biol. 2006; 10: 445–452. doi: 10.1016/j.cbpa.2006.08.018 16935548 10.1016/j.cbpa.2006.08.018

[11] Paravastu AK, Leapman RD, Yau W-M, Tycko R. Molecular structural basis for polymorphism in Alzheimer's β-amyloid fibrils. Proc Natl Acad Sci U S A. 2008; 105: 18349–18354. doi: 10.1073/pnas.0806270105 19015532 10.1073/pnas.0806270105 PMC2587602

[12] Lu J-X, Qiang W, Yau W-M, Schwieters Charles D, Meredith Stephen C, Tycko R. Molecular structure of β-amyloid fibrils in Alzheimer’s disease brain tissue. Cell. 2013; 154: 1257–1268. doi: 10.1016/j.cell.2013.08.035 24034249 10.1016/j.cell.2013.08.035 PMC3814033

[13] Gremer L, Schölzel D, Schenk C, Reinartz E, Labahn J, Ravelli RBG, et al Fibril structure of amyloid-β(1–42) by cryoelectron microscopy. Science. 2017 doi: 10.1126/science.aao2825 28882996 10.1126/science.aao2825 PMC6080689

[14] Liu R, McAllister C, Lyubchenko Y, Sierks MR. Residues 17–20 and 30–35 of beta-amyloid play critical roles in aggregation. J Neurosci Res. 2004; 75: 162–171. doi: 10.1002/jnr.10859 14705137 10.1002/jnr.10859

[15] Spencer RK, Li H, Nowick JS. X-ray crystallographic structures of trimers and higher-order oligomeric assemblies of a peptide derived from Aβ17–36. J Am Chem Soc. 2014; 136: 5595–5598. doi: 10.1021/ja5017409 24669800 10.1021/ja5017409 PMC4004244

[16] Lendel C, Bjerring M, Dubnovitsky A, Kelly RT, Filippov A, Antzutkin ON, et al A hexameric peptide barrel as building block of amyloid-β protofibrils. Angew Chem Int Ed. 2014; 53: 12756–12760. 10.1002/anie.201406357 25256598

[17] Larini L, Shea J-E. Role of β-hairpin formation in aggregation: the self-assembly of the amyloid-β(25–35) peptide. Biophys J. 2012; 103: 576–586. doi: 10.1016/j.bpj.2012.06.027 22947874 10.1016/j.bpj.2012.06.027 PMC3414875

[18] Ma B, Nussinov R. Stabilities and conformations of Alzheimer's β-amyloid peptide oligomers (Aβ16–22, Aβ16–35, and Aβ10–35): Sequence effects. Proc Natl Acad Sci U S A. 2002; 99: 14126–14131. doi: 10.1073/pnas.212206899 12391326 10.1073/pnas.212206899 PMC137848

[19] Burkoth TS, Benzinger TLS, Urban V, Morgan DM, Gregory DM, Thiyagarajan P, et al Structure of the β-amyloid(10–35) fibril. J Am Chem Soc. 2000; 122: 7883–7889.

[20] Do TD, LaPointe NE, Nelson R, Krotee P, Hayden EY, Ulrich B, et al Amyloid β-protein C-terminal fragments: Formation of cylindrins and β-barrels. J Am Chem Soc. 2016; 138: 549–557. doi: 10.1021/jacs.5b09536 26700445 10.1021/jacs.5b09536 PMC4741107

[21] Balbach JJ, Ishii Y, Antzutkin ON, Leapman RD, Rizzo NW, Dyda F, et al Amyloid fibril formation by Aβ16–22, a seven-residue fragment of the Alzheimer's β-amyloid peptide, and structural characterization by solid state NMR. Biochemistry. 2000; 39: 13748–13759. 11076514 10.1021/bi0011330

[22] Lu K, Jacob J, Thiyagarajan P, Conticello VP, Lynn DG. Exploiting amyloid fibril lamination for nanotube self-assembly. J Am Chem Soc. 2003; 125: 6391–6393. doi: 10.1021/ja0341642 12785778 10.1021/ja0341642

[23] Gnanakaran S, Nussinov R, García AE. Atomic-level description of amyloid β-dimer formation. J Am Chem Soc. 2006; 128: 2158–2159. doi: 10.1021/ja0548337 16478138 10.1021/ja0548337

[24] Nguyen PH, Li MS, Stock G, Straub JE, Thirumalai D. Monomer adds to preformed structured oligomers of Aβ-peptides by a two-stage dock–lock mechanism. Proc Natl Acad Sci U S A. 2007; 104: 111–116. doi: 10.1073/pnas.0607440104 17190811 10.1073/pnas.0607440104 PMC1766316

[25] Colletier J-P, Laganowsky A, Landau M, Zhao M, Soriaga AB, Goldschmidt L, et al Molecular basis for amyloid-β polymorphism. Proc Natl Acad Sci U S A. 2011; 108: 16938–16943. doi: 10.1073/pnas.1112600108 21949245 10.1073/pnas.1112600108 PMC3193189

[26] Cheng P-N, Liu C, Zhao M, Eisenberg D, Nowick JS. Amyloid β-sheet mimics that antagonize protein aggregation and reduce amyloid toxicity. Nat Chem. 2012; 4: 927–933. doi: 10.1038/nchem.1433 23089868 10.1038/nchem.1433 PMC3481199

[27] Liu C, Zhao M, Jiang L, Cheng P-N, Park J, Sawaya MR, et al Out-of-register β-sheets suggest a pathway to toxic amyloid aggregates. Proc Natl Acad Sci U S A. 2012; 109: 20913–20918. doi: 10.1073/pnas.1218792109 23213214 10.1073/pnas.1218792109 PMC3529048

[28] Irbäck A, Mitternacht S. Spontaneous β-barrel formation: An all-atom Monte Carlo study of Aβ16–22 oligomerization. Proteins: Struct, Funct, Bioinf. 2008; 71: 207–214. 10.1002/prot.21682 17932914

[29] Xie L, Luo Y, Wei G. Aβ(16–22) peptides can assemble into ordered β-barrels and bilayer β-sheets, while substitution of phenylalanine 19 by tryptophan increases the population of disordered aggregates. J Phys Chem B. 2013; 117: 10149–10160. doi: 10.1021/jp405869a 23926957 10.1021/jp405869a

[30] Fossati S, Cam J, Meyerson J, Mezhericher E, Romero IA, Couraud PO, et al Differential activation of mitochondrial apoptotic pathways by vasculotropic amyloid-β variants in cells composing the cerebral vessel walls. FASEB J. 2010; 24: 229–241. doi: 10.1096/fj.09-139584 19770225 10.1096/fj.09-139584 PMC2797039

[31] Panda PK, Patil AS, Patel P, Panchal H. Mutation-based structural modification and dynamics study of amyloid beta peptide (1–42): An in-silico-based analysis to cognize the mechanism of aggregation. Genomics Data. 2016; 7: 189–194. doi: 10.1016/j.gdata.2016.01.003 26981406 10.1016/j.gdata.2016.01.003 PMC4778649

[32] Brunelle P, Rauk A. The radical model of Alzheimer's disease: Specific recognition of Gly29 and Gly33 by Met35 in a β-sheet model of Aβ: An ONIOM study. J Alzheimer's Dis. 2002; 4: 283–289. 12446930 10.3233/jad-2002-4403

[33] Kanski J, Varadarajan S, Aksenova M, Butterfield DA. Role of glycine-33 and methionine-35 in Alzheimer’s amyloid β-peptide 1–42-associated oxidative stress and neurotoxicity. Biochim Biophys Acta, Mol Basis Dis. 2002; 1586: 190–198. 10.1016/s0925-4439(01)00097-7 11959460

[34] Vignaud H, Bobo C, Lascu I, Sörgjerd KM, Zako T, Maeda M, et al A structure-toxicity study of Aß42 reveals a new anti-parallel aggregation pathway. PLoS One. 2013; 8: e80262 doi: 10.1371/journal.pone.0080262 24244667 10.1371/journal.pone.0080262 PMC3823702

[35] Henry S, Vignaud H, Bobo C, Decossas M, Lambert O, Harte E, et al Interaction of Aβ1–42 amyloids with lipids promotes “off-pathway” oligomerization and membrane damage. Biomacromolecules. 2015; 16: 944–950. doi: 10.1021/bm501837w 25689632 10.1021/bm501837w

[36] Bonhommeau S, Talaga D, Hunel J, Cullin C, Lecomte S. Tip-enhanced Raman spectroscopy to distinguish toxic oligomers from Aβ1–42 fibrils at the nanometer scale. Angew Chem. 2017; 129: 1797–1800. 10.1002/anie.201610399 28071842

[37] Van Der Spoel D, Lindahl E, Hess B, Groenhof G, Mark AE, Berendsen HJC. GROMACS: Fast, flexible, and free. J Comput Chem. 2005; 26: 1701–1718. doi: 10.1002/jcc.20291 16211538 10.1002/jcc.20291

[38] Oostenbrink C, Villa A, Mark AE, Van Gunsteren WF. A biomolecular force field based on the free enthalpy of hydration and solvation: The GROMOS force-field parameter sets 53A5 and 53A6. J Comput Chem. 2004; 25: 1656–1676. doi: 10.1002/jcc.20090 15264259 10.1002/jcc.20090

[39] Zhou X, Xi W, Luo Y, Cao S, Wei G. Interactions of a water-soluble fullerene derivative with amyloid-β protofibrils: dynamics, binding mechanism, and the resulting salt-bridge disruption. J Phys Chem B. 2014; 118: 6733–6741. doi: 10.1021/jp503458w 24857343 10.1021/jp503458w

[40] Xie L, Luo Y, Lin D, Xi W, Yang X, Wei G. The molecular mechanism of fullerene-inhibited aggregation of Alzheimer's β-amyloid peptide fragment. Nanoscale. 2014; 6: 9752–9762. doi: 10.1039/c4nr01005a 25004796 10.1039/c4nr01005a

[41] Krone MG, Hua L, Soto P, Zhou R, Berne BJ, Shea J-E. Role of water in mediating the assembly of Alzheimer amyloid-β Aβ16−22 protofilaments. J Am Chem Soc. 2008; 130: 11066–11072. doi: 10.1021/ja8017303 18661994 10.1021/ja8017303 PMC3066469

[42] Du W-J, Guo J-J, Gao M-T, Hu S-Q, Dong X-Y, Han Y-F, et al Brazilin inhibits amyloid β-protein fibrillogenesis, remodels amyloid fibrils and reduces amyloid cytotoxicity. Sci Rep. 2015; 5: 7992 doi: 10.1038/srep07992 25613018 10.1038/srep07992 PMC4303869

[43] Olubiyi OO, Strodel B. Structures of the amyloid β-peptides Aβ1–40 and Aβ1–42 as influenced by pH and a D-peptide. J Phys Chem B. 2012; 116: 3280–3291. doi: 10.1021/jp2076337 22300010 10.1021/jp2076337

[44] Berendsen HJC, Postma JPM, van Gunsteren WF, Hermans J (1981) Interaction models for water in relation to protein hydration In: Pullman B, editor. Intermolecular Forces: Springer Netherlands pp. 331–342.

[45] Sugita Y, Okamoto Y. Replica-exchange molecular dynamics method for protein folding. Chem Phys Lett. 1999; 314: 141–151.

[46] Okamoto Y. Generalized-ensemble algorithms: enhanced sampling techniques for Monte Carlo and molecular dynamics simulations. J Mol Graphics Modell. 2004; 22: 425–439. 10.1016/j.jmgm.2003.12.009 15099838

[47] Nadler W, Hansmann UHE. Optimized explicit-solvent replica exchange molecular dynamics from scratch. J Phys Chem B. 2008; 112: 10386–10387. doi: 10.1021/jp805085y 18671362 10.1021/jp805085y

[48] Hess B, Bekker H, Berendsen HJC, Fraaije JGEM. LINCS: A linear constraint solver for molecular simulations. J Comput Chem. 1997; 18: 1463–1472.

[49] Miyamoto S, Kollman PA. Settle: An analytical version of the SHAKE and RATTLE algorithm for rigid water models. J Comput Chem. 1992; 13: 952–962.

[50] Bussi G, Donadio D, Parrinello M. Canonical sampling through velocity rescaling. J Chem Phys. 2007; 126: 014101 doi: 10.1063/1.2408420 17212484 10.1063/1.2408420

[51] Parrinello M, Rahman A. Polymorphic transitions in single crystals: A new molecular dynamics method. J Appl Phys. 1981; 52: 7182–7190.

[52] Nosé S, Klein ML. Constant pressure molecular dynamics for molecular systems. Mol Phys. 1983; 50: 1055–1076.

[53] Essmann U, Perera L, Berkowitz ML, Darden T, Lee H, Pedersen LG. A smooth particle mesh Ewald method. J Chem Phys. 1995; 103: 8577–8593.

[54] Daura X, Gademann K, Jaun B, Seebach D, van Gunsteren WF, Mark AE. Peptide folding: when simulation meets experiment. Angew Chem Int Ed. 1999; 38: 236–240.

[55] Kabsch W, Sander C. Dictionary of protein secondary structure: pattern recognition of hydrogen-bonded and geometrical features. Biopolymers. 1983; 22: 2577–2637. doi: 10.1002/bip.360221211 6667333 10.1002/bip.360221211

[56] Truex NL, Wang Y, Nowick JS. Assembly of peptides derived from β-sheet regions of β-amyloid. J Am Chem Soc. 2016; 138: 13882–13890. doi: 10.1021/jacs.6b06000 27642651 10.1021/jacs.6b06000 PMC5089065

[57] Lei J, Qi R, Wei G, Nussinov R, Ma B. Self-aggregation and coaggregation of the p53 core fragment with its aggregation gatekeeper variant. Phys Chem Chem Phys. 2016; 18: 8098–8107. doi: 10.1039/c5cp06538k 26923710 10.1039/c5cp06538k PMC6456058

[58] Lu Y, Wei G, Derreumaux P. Effects of G33A and G33I mutations on the structures of monomer and dimer of the amyloid-β fragment 29−42 by replica exchange molecular dynamics simulations. J Phys Chem B. 2011; 115: 1282–1288. doi: 10.1021/jp110269a 21186801 10.1021/jp110269a

[59] Laganowsky A, Liu C, Sawaya MR, Whitelegge JP, Park J, Zhao M, et al Atomic view of a toxic amyloid small oligomer. Science. 2012; 335: 1228–1231. doi: 10.1126/science.1213151 22403391 10.1126/science.1213151 PMC3959867

[60] Berhanu WM, Hansmann UHE. The stability of cylindrin β-barrel amyloid oligomer models–A molecular dynamics study. Proteins: Struct, Funct, Bioinf. 2013; 81: 1542–1555. 10.1002/prot.24302 PMC4206217 23606599

[61] Jang H, Zheng J, Lal R, Nussinov R. New structures help the modeling of toxic amyloidβ ion channels. Trends Biochem Sci. 2008; 33: 91–100. doi: 10.1016/j.tibs.2007.10.007 18182298 10.1016/j.tibs.2007.10.007

[62] Jang H, Arce FT, Ramachandran S, Capone R, Lal R, Nussinov R. β-Barrel topology of Alzheimer's β-amyloid ion channels. J Mol Biol. 2010; 404: 917–934. doi: 10.1016/j.jmb.2010.10.025 20970427 10.1016/j.jmb.2010.10.025 PMC7291702

[63] Chang Z, Luo Y, Zhang Y, Wei G. Interactions of Aβ25−35 β-barrel-like oligomers with anionic lipid bilayer and resulting membrane leakage: An all-atom molecular dynamics study. J Phys Chem B. 2011; 115: 1165–1174. doi: 10.1021/jp107558e 21192698 10.1021/jp107558e

[64] Zhang H, Xi W, Hansmann UHE, Wei Y. Fibril–barrel transitions in cylindrin amyloids. J Chem Theory Comput. 2017; 13: 3936–3944. doi: 10.1021/acs.jctc.7b00383 28671829 10.1021/acs.jctc.7b00383

[65] Stroud JC, Liu C, Teng PK, Eisenberg D. Toxic fibrillar oligomers of amyloid-β have cross-β structure. Proc Natl Acad Sci U S A. 2012; 109: 7717–7722. doi: 10.1073/pnas.1203193109 22547798 10.1073/pnas.1203193109 PMC3356606

[66] Xi W, Wang W, Abbott G, Hansmann UHE. Stability of a recently found triple-β-stranded Aβ1–42 fibril motif. J Phys Chem B. 2016; 120: 4548–4557. doi: 10.1021/acs.jpcb.6b01724 27137996 10.1021/acs.jpcb.6b01724

[67] Wang J, Tao K, Zhou P, Pambou E, Li Z, Xu H, et al Tuning self-assembled morphology of the Aβ(16–22) peptide by substitution of phenylalanine residues. Colloids Surf, B. 2016; 147: 116–123. 10.1016/j.colsurfb.2016.07.052 27497075

[68] Jang H, Arce FT, Ramachandran S, Capone R, Azimova R, Kagan BL, et al Truncated β-amyloid peptide channels provide an alternative mechanism for Alzheimer’s Disease and Down syndrome. Proc Natl Acad Sci U S A. 2010; 107: 6538–6543. doi: 10.1073/pnas.0914251107 20308552 10.1073/pnas.0914251107 PMC2851998

[69] Lu Y, Derreumaux P, Guo Z, Mousseau N, Wei G. Thermodynamics and dynamics of amyloid peptide oligomerization are sequence dependent. Proteins: Struct, Funct, Bioinf. 2009; 75: 954–963. 10.1002/prot.22305 19089954

[70] Chouchane K, Vendrely C, Amari M, Moreaux K, Bruckert F, Weidenhaupt M. Dual effect of (LK)nL peptides on the onset of insulin amyloid fiber formation at hydrophobic surfaces. J Phys Chem B. 2015; 119: 10543–10553. doi: 10.1021/acs.jpcb.5b07365 26234630 10.1021/acs.jpcb.5b07365

[71] Latshaw David C, II, Hall Carol K. Effects of hydrophobic macromolecular crowders on amyloid β (16–22) aggregation. Biophys J. 2015; 109: 124–134. doi: 10.1016/j.bpj.2015.05.032 26153709 10.1016/j.bpj.2015.05.032 PMC4571038

[72] Harmeier A, Wozny C, Rost BR, Munter L-M, Hua H, Georgiev O, et al Role of amyloid-β glycine 33 in oligomerization, toxicity, and neuronal plasticity. J Neurosci. 2009; 29: 7582–7590. doi: 10.1523/JNEUROSCI.1336-09.2009 19515926 10.1523/JNEUROSCI.1336-09.2009 PMC6665404

[73] Berhanu WM, Hansmann UHE. Side-chain hydrophobicity and the stability of Aβ16–22 aggregates. Protein Sci. 2012; 21: 1837–1848. doi: 10.1002/pro.2164 23015407 10.1002/pro.2164 PMC3575914

[74] Berhanu WM, Hansmann UHE (2014) Chapter four—Stability of amyloid oligomers In: Tatyana K-C, editor. Advances in Protein Chemistry and Structural Biology: Academic Press pp. 113–141. 10.1016/bs.apcsb.2014.06.006 25443956

[75] Xie L, Lin D, Luo Y, Li H, Yang X, Wei G. Effects of hydroxylated carbon nanotubes on the aggregation of Aβ16–22 peptides: A combined simulation and experimental study. Biophys J. 2014; 107: 1930–1938. doi: 10.1016/j.bpj.2014.08.034 25418174 10.1016/j.bpj.2014.08.034 PMC4213673

