# Supplementary material for: Assemblies of amyloid-β30–36 hexamer and its G33V/L34T mutants by replica-exchange molecular dynamics simulation
Source: PLoS One. 2017 Nov 29;12(11):e0188794. doi: 10.1371/journal.pone.0188794 (PMC5706729; doi:10.1371/journal.pone.0188794)
Supplement: S1 Text — (PDF) [file pone.0188794.s001.pdf]

Supplementary simulation details for:

Assemblies of Amyloid-$\beta_{30\text{-}36}$ Hexamer and Its G33V/L34T Mutants by Replica-Exchange Molecular Dynamics Simulation

Zhenyu Qian[1*], Qingwen Zhang[2], Yu Liu[1], Peijie Chen[1*]

1 Key Laboratory of Exercise and Health Sciences (Ministry of Education) and School of Kinesiology, Shanghai University of Sport, Shanghai, China
2 College of Physical Education and Training, Shanghai University of Sport, Shanghai, China

* Corresponding author
E-mail: qianzhenyu@sus.edu.cn (ZQ); chenpeijie@sus.edu.cn (PC)

Simulation details

The peptides for WT, G33V and L34T A$\beta_{30\text{-}36}$ hexamer systems are placed randomly in the simulation box, and undergo a 2ns pre-simulation at 500K to make the peptides random coil. The REMD simulations of the three systems include 40 replicas, and the temperatures were varied from 305 K to 430 K (temperature list: 305.00, 307.70, 310.42, 313.17, 315.94, 318.73, 321.55, 324.39, 327.26, 330.16, 333.08, 336.03, 339.00, 342.00, 345.02, 348.07, 351.15, 354.26, 357.39, 360.56, 363.74, 366.96, 370.21, 373.48, 376.79, 380.12, 383.48, 386.87, 390.30, 393.75, 397.23, 400.75, 404.29, 407.87, 411.48, 415.12, 418.79, 422.49, 426.23, 430.00 K).

The free energy surfaces (or potential of mean force, PMF) were constructed using the relation $-RT\ln H(x, y)$, where $H(x, y)$ is the histogram of two selected reaction coordinates, x and y. In this study, the x coordinate is the number of hydrogen bonds (H-bonds) and the y coordinate is the radius of gyration of the Aβ peptides. The chain-independent Cα-root mean square deviation (Cα-RMSD) is calculated by completely neglecting the chain identifier in the coordinate file to obtain the smallest RMSD as the chains are topologically identical. The inter/intra chain interactions were estimated by the probability of residue-residue contacts. A contact is defined when the aliphatic carbon atoms of two non-sequential main chains (or side chains) are located within 0.54 nm or any other atoms of two non-sequential main chains (or side chains) within 0.46 nm. The end-to-end distance is calculated from the A30 Cα atom to the V36 Cα atom of each chain, and one hexamer has six values of end-to-end distances. For each system, we counted all the end-to-end distance values and got its probability density function. The radius of gyration (RG) is calculated through the three-dimensional coordinate of all the Cα atoms of a hexamer, and one hexamer has one value of RG as an average.

Convergence check of the REMD runs

The probability density function (PDF) of end-to-end distance for all chains, number of H-bonds, RG, and solvent accessible surface area (SASA), are shown in Figure S2. The PDF and propensity of the REMD runs in two independent time intervals of 50-100 ns and 100-150 ns are almost the same, which suggests the REMD simulations are reasonably converged. The secondary structure contents for three systems are shown in Figure S3, which are quite similar within two independent time intervals. Thus our analyses are based on the last 100ns data.
