# Supplementary figures and images for: Assemblies of amyloid-β30–36 hexamer and its G33V/L34T mutants by replica-exchange molecular dynamics simulation
Source: PLoS One. 2017 Nov 29;12(11):e0188794. doi: 10.1371/journal.pone.0188794 (PMC5706729; doi:10.1371/journal.pone.0188794)

(a) WT

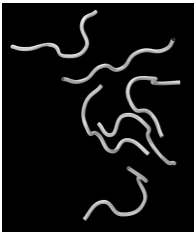

(b) G33V

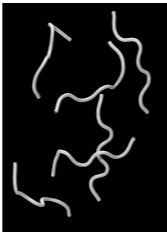

(c) L34T

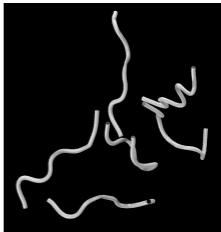

Supplement: S1 Fig — The initial structures of WT (a), G33V (b) and L34T (c) Aβ30–36 hexamer for REMD simulation. The peptides are in cartoon representation. (PDF) [file pone.0188794.s002.pdf]

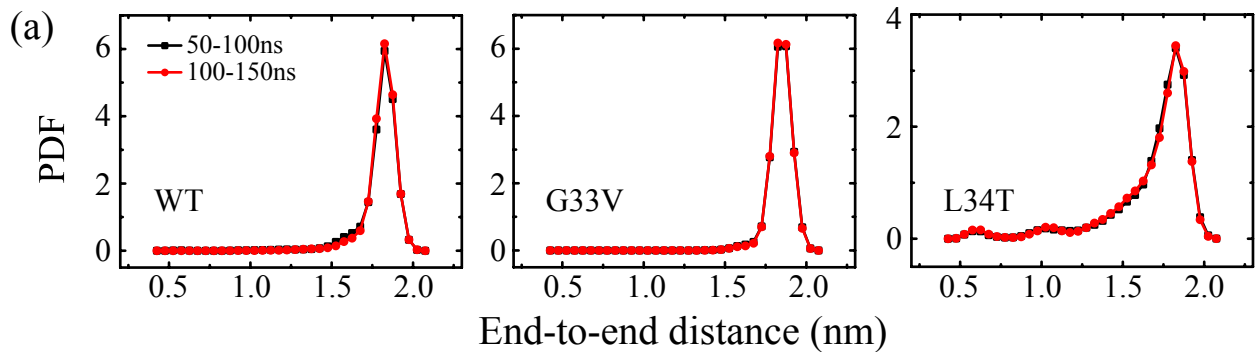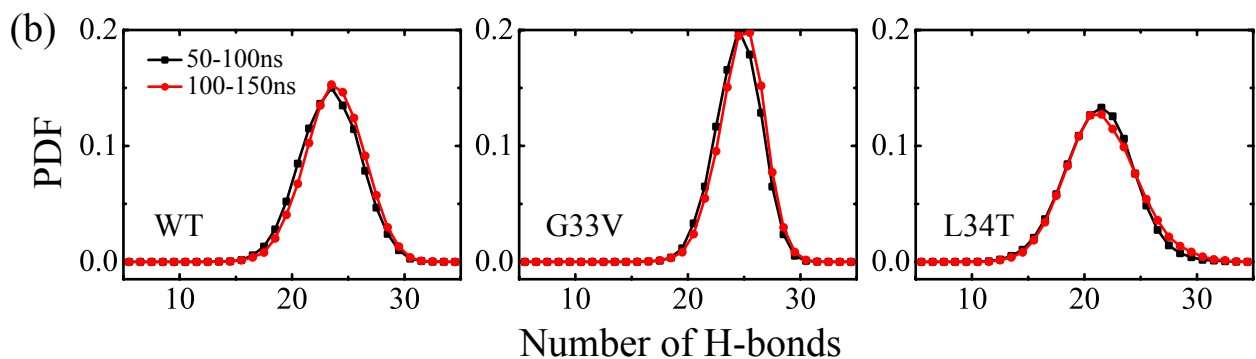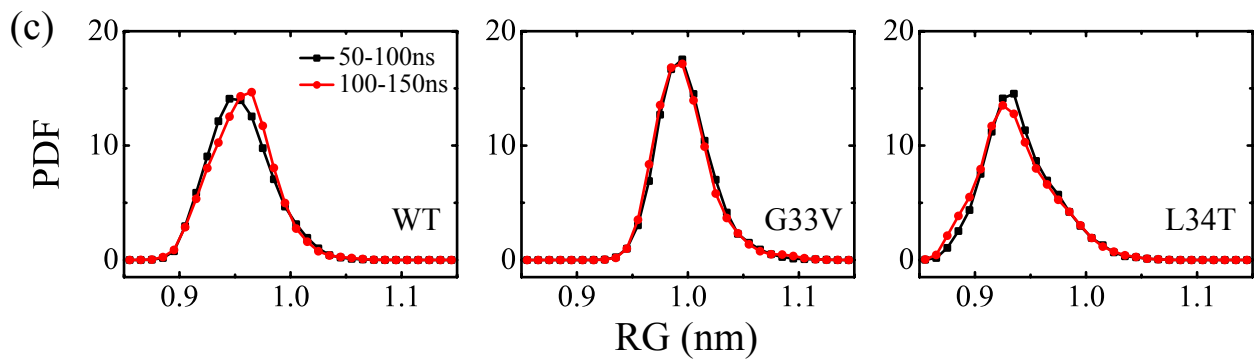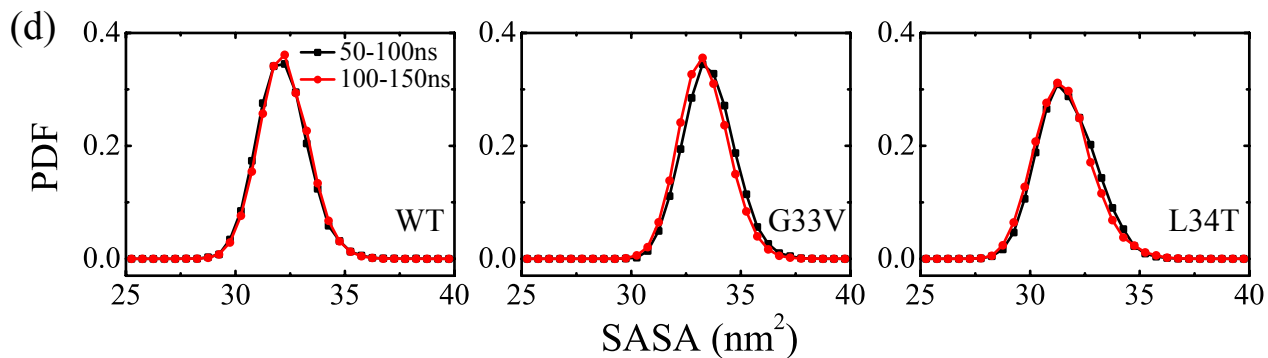

Supplement: S2 Fig — The probability density function (PDF) of end-to-end distance for all chains (a), number of H-bonds (b), radius of gyration (RG) (c), and solvent accessible surface area (SASA) (d) for three systems within two independent time intervals of 50–100 ns and 100–150 ns at 310K. (PDF) [file pone.0188794.s003.pdf]

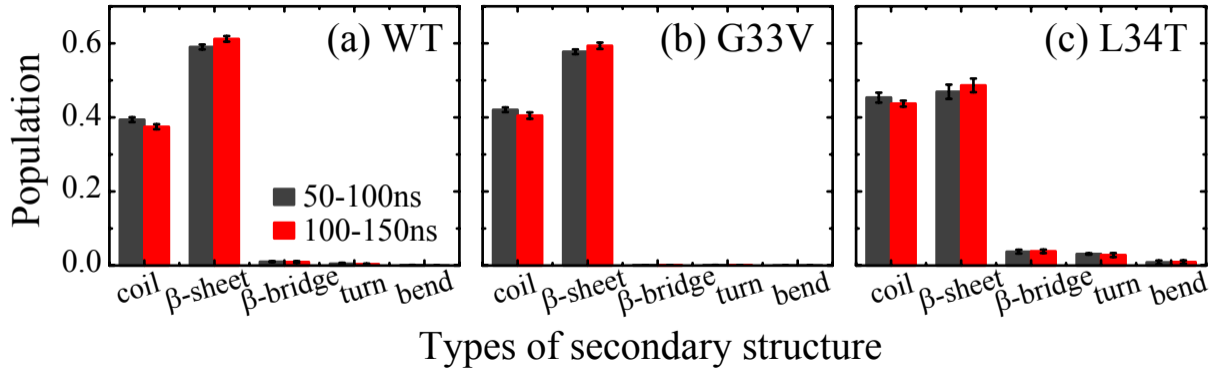

Supplement: S3 Fig — (PDF) [file pone.0188794.s004.pdf]

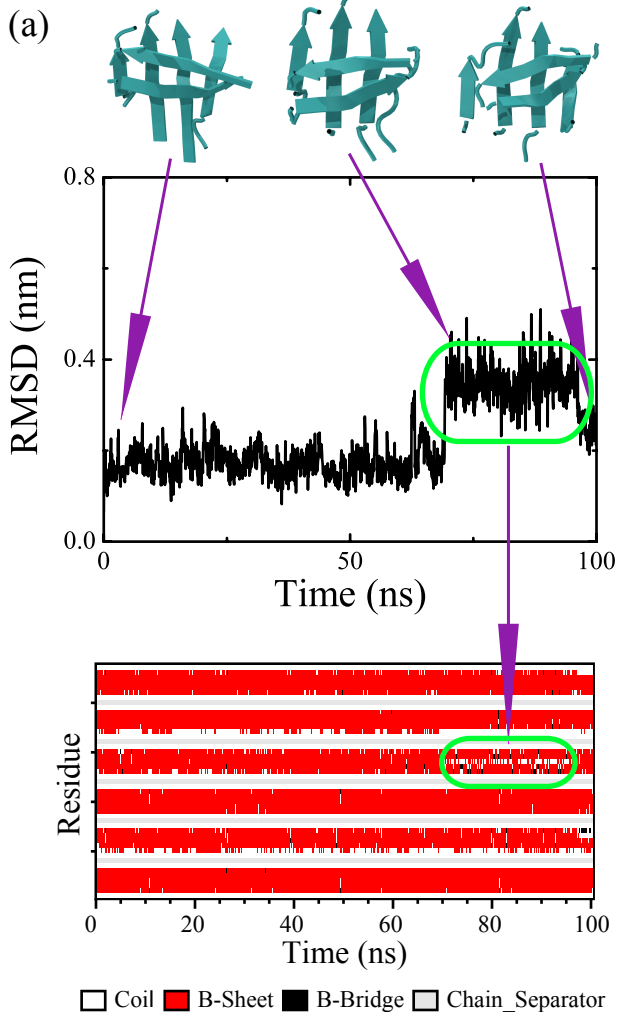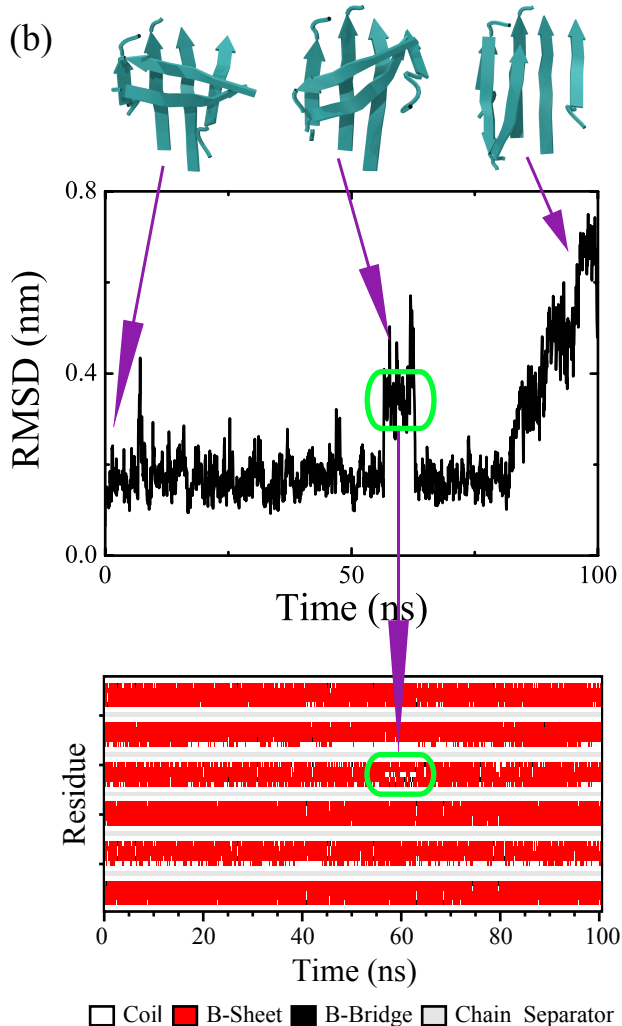

Supplement: S4 Fig — Both conventional MD simulations are initiated from the 4 + 2 β-sheet bilayer in Cluster-2: (a) the peptides transfer to a closed barrel-like structure; (b) the two-stranded bilayer drifts away from the four-stranded bilayer. (PDF) [file pone.0188794.s005.pdf]

(a)  $\text{dis\_ete}=5.77 \text{ \AA}$

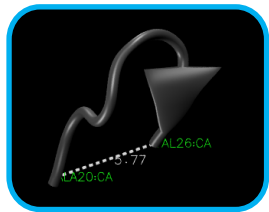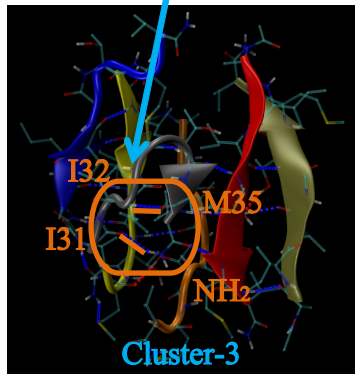

(b)  $\text{dis\_ete}=9.66 \text{ \AA}$

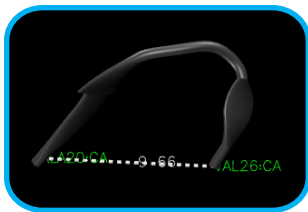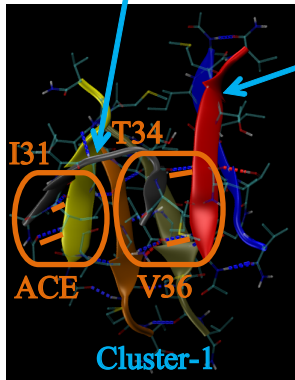

(c)  $\text{dis\_ete}=17.90 \text{ \AA}$

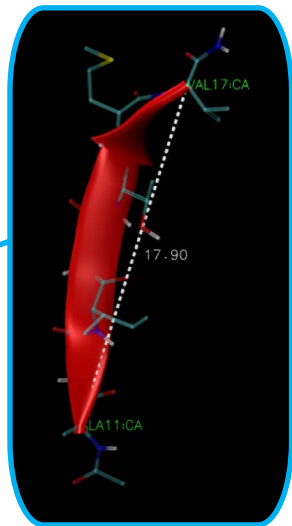

Supplement: S6 Fig — Snapshots of one L34T mutant Aβ30–36 peptide with an end-to-end distance of 5.77 Å (a), 9.66 Å (b) and 17.90 Å (c). The pane-contained H-bonds are highlighted in orange, with explicit names of the residues involved in H-bonding. The end-to-end distance is calculated from the A30 Cα atom to the V36 Cα atom. The secondary structures are shown in cartoon representation, and the peptides in licorice representation with carbon atoms in cyan, oxygen atoms in red, nitrogen atoms in blue, sulfur atoms in yellow and hydrogen atoms in white. (PDF) [file pone.0188794.s007.pdf]

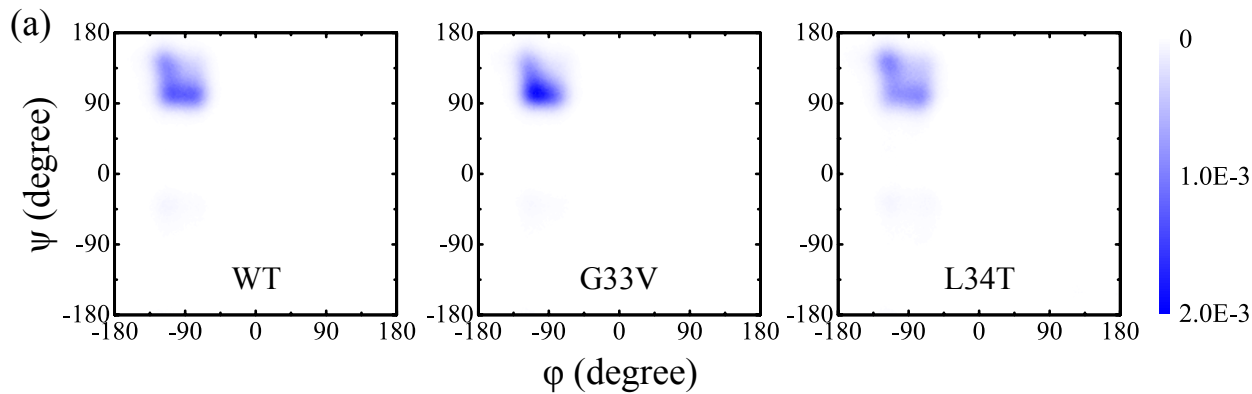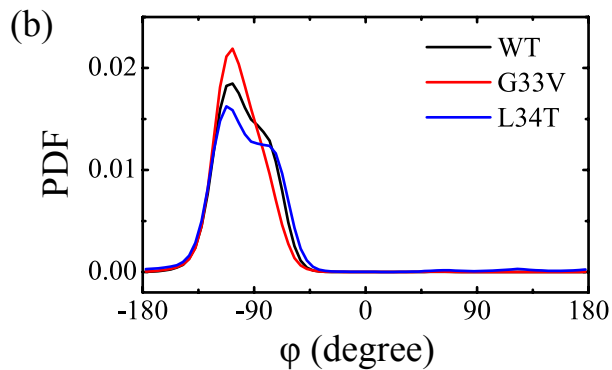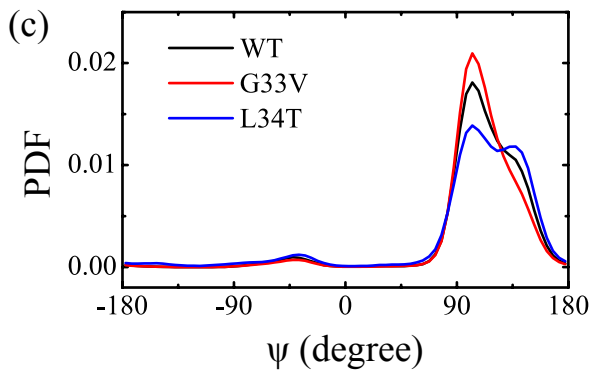

Supplement: S7 Fig — The distribution of dihedral angles of the first eight most-populated conformations for WT, G33V and L34T Aβ30–36 hexamers at 310K: (a) the probability in dihedral angle φ-ψ plane; (b) PDF of φ and ψ. (PDF) [file pone.0188794.s008.pdf]

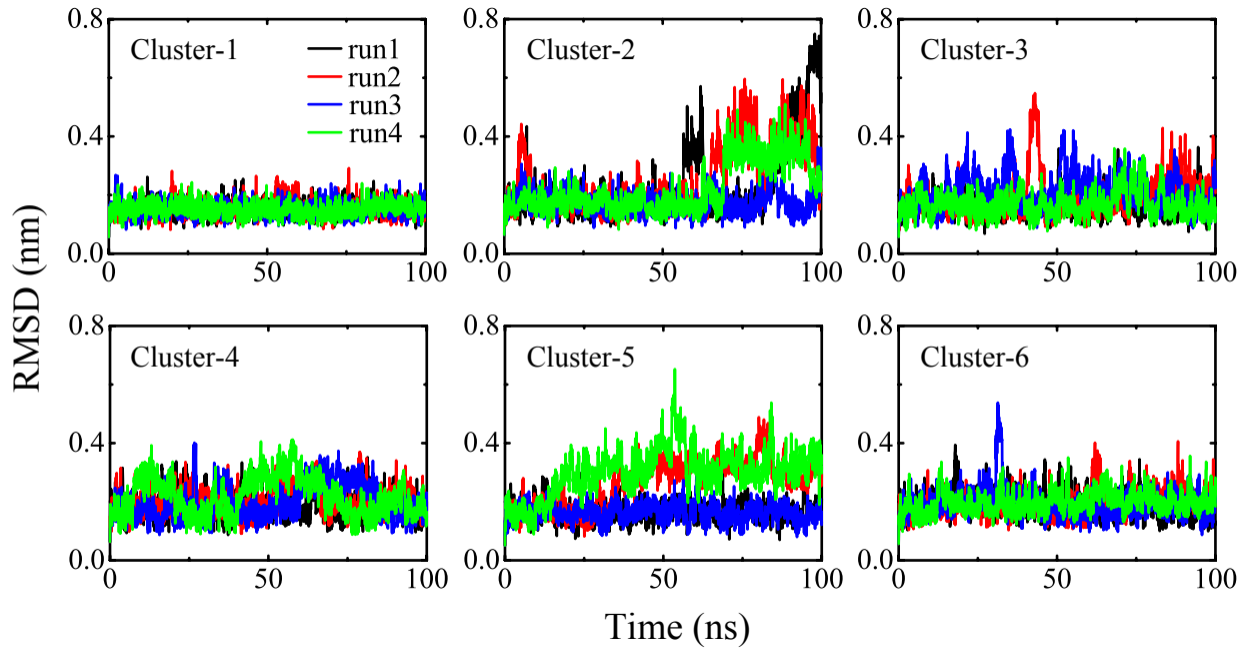

Supplement: S8 Fig — These conventional MD simulations are initiated from the conformations in the first six most-populated clusters. Different colors represent independent MD runs. The green line of Cluster-2 corresponds to a transformation from a 4 + 2 β-sheet bilayer to a closed barrel-like structure. (PDF) [file pone.0188794.s009.pdf]

PDF

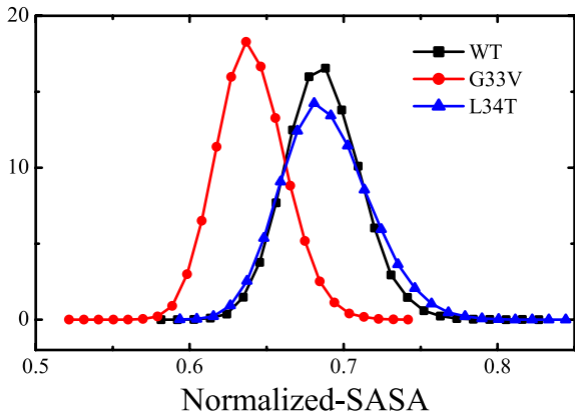

Supplement: S9 Fig — (PDF) [file pone.0188794.s010.pdf]
