# Supplementary material for: Assemblies of amyloid-β30–36 hexamer and its G33V/L34T mutants by replica-exchange molecular dynamics simulation
Source: PLoS One. 2017 Nov 29;12(11):e0188794. doi: 10.1371/journal.pone.0188794 (PMC5706729; doi:10.1371/journal.pone.0188794)
Supplement: S5 Fig — The color indicates the average number of H-bonds. AP0/P0 represents in-register antiparallel/parallel β-sheets; P1/P2 represents 1-residue-shift/2-residue-shift out-of-register parallel β-sheets. (PDF) [file pone.0188794.s006.pdf]

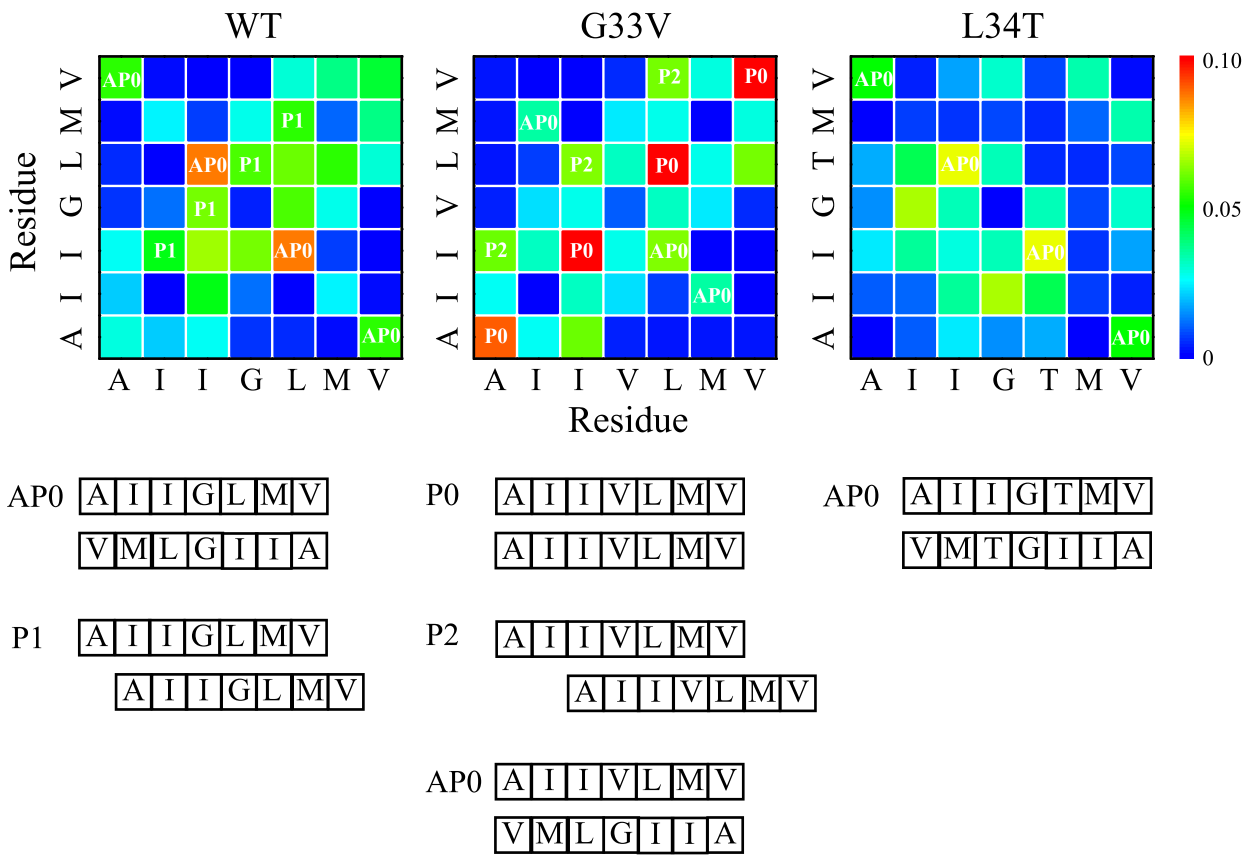

WT
G33V
L34T
Residue
A I I G L M V
A I I V L M V
A I I G T M V
Residue
0.10
0.05
0
AP0
P1
P0
P2
AP0 A I I G L M V
V M L G I I A
P1 A I I G L M V
A I I G L M V
P0 A I I V L M V
A I I V L M V
P2 A I I V L M V
A I I V L M V
AP0 A I I V L M V
V M L G I I A
AP0 A I I G T M V
V M T G I I A
